# Integrated analysis of M2 macrophage-related gene prognostic model and single-cell sequence to predict immunotherapy response in lung adenocarcinoma

**DOI:** 10.3389/fgene.2025.1519677

**Published:** 2025-02-03

**Authors:** Meifang Li, Zhiping Wang, Bin Huang, Yanyun Lai, Meng Zhang, Cheng Lin

**Affiliations:** ^1^ Department of Medical Oncology, Fujian Cancer Hospital, Clinical Oncology School of Fujian Medical University, Fuzhou, China; ^2^ Department of Radiation Oncology, Fujian Cancer Hospital, Clinical Oncology School of Fujian Medical University, Fuzhou, China

**Keywords:** lung adenocarcinoma, tumor microenvironment, macrophages, prognosis, immunotherapy

## Abstract

**Background:**

Lung adenocarcinoma (LUAD) patients have high heterogeneity. The significance and clinical value of M2 macrophage-related genes in LUAD require further exploration. We aimed to construct a prognostic signature to predict the immunotherapy efficacy and prognosis in LUAD.

**Methods:**

GSE26939 and GSE19188 chips were downloaded from the Gene Expression Omnibus (GEO). Weighted gene co-expression network analysis (WGCNA) and least absolute shrinkage and selection operator (LASSO) analysis were used to screen M2 macrophage-related prognostic genes. A signature based on M2 macrophage-related prognostic genes was established and used to predict the prognosis and immunotherapy efficacy in LUAD.

**Results:**

Twenty-two M2 macrophage-related genes associated with the prognosis of LUAD were confirmed using WGCNA, and then two molecular subtypes were identified with significantly different survival, gene expressions, and clinic characteristics were classified. LASSO analysis identified nine M2 macrophage-related prognostic genes to establish a risk signature, classifying patients into low- and high-risk groups. Data indicated that low-risk patients had better survival. Moreover, the signature was an independent prognostic factor for LUAD and a potential biomarker for patients receiving immunotherapy. Single-cell transcriptome analysis may provide important information on molecular subtypes and heterogeneity.

**Conclusion:**

Risk signature based on M2 macrophage-related genes is a valuable tool for predicting prognosis and immunotherapy response in patients with LUAD.

## 1 Introduction

Lung adenocarcinoma (LUAD) is the primary subtype of non-small cell lung cancer (NSCLC), and accounts for more than 50% of all NSCLC cases. The 5-year survival for patients with advanced LUAD is lower than 20% (Asamura et al., 2023). In recent years, the emergence of immune checkpoint inhibitors (ICIs) and targeted drugs has completely changed the outcomes of advanced LUAD (Liu et al., 2023; The Lancet, 2024). However, treatment unresponsiveness and drug resistance are common, especially in immunotherapy (Mogavero et al., 2023). The poor curative effects largely stem from the complicated molecular features caused by the high heterogeneity of LUAD. Therefore, the exploration of meaningful “signatures” to predict the prognosis and assist the management of LUAD is urgently needed.

Many clinical and molecular factors influence the efficacy of ICIs (Thummalapalli et al., 2023). Thus, exploration of cellular and molecular mechanisms, thus assisting in achieving durable responses to ICIs is essential. Tumor microenvironment (TME), including tumor cells, immune cells, stromal cells, and extracellular matrix (ECM), as well as driver genes and other genes, are involved in the treatment response and prognosis in a variety of cancers (Binnewies et al., 2018). At present, attention is focused on the clinical significance of T cells in TME. KEYNOTE-028 trail revealed that the T-cell-inflamed gene-expression profile (TcellinfGEP) could predict response to pembrolizumab in 20 tumor types (Ott et al., 2019), which was also demonstrated in advanced NSCLC in KEYNOTE-494/KeyImPaCT trail (Gutierrez et al., 2023). Of note, other immune cells, like cancer-associated fibroblast (CAF) and tumor-associated macrophages (TAM), were also reported to be closely associated with the development of NSCLC (Cords et al., 2024; Zhang et al., 2023). However, the values of TAM in LUAD are still unclear in clinical practice since TAM was supposed to be a double-edged sword in the TME.

Macrophages can be polarized into M1 and M2 types under different microenvironments and stimulators (Funes et al., 2018). The function of TAM is similar to M2-like macrophages in cancers (Sarode et al., 2020; Sumitomo et al., 2019; Xu et al., 2020). M2 TAMs can promote cancer proliferation, invasion, migration, angiogenesis, and multidrug resistance. More importantly, TAMs can inhibit the activation and aggregation of immune cells by secreting cytokines and chemokines, establishing suppressive TME. Therefore, in-depth research on the role of M2 macrophage in LUAD and the construction of a prognostic signature associated with M2 macrophage are necessary.

In this study, we sought to screen an M2 macrophage-related signature and to predict the prognosis and immunotherapy efficacy of LUAD patients. We found that an M2 macrophage-related signature based on characteristic genes was a novel biomarker in the management of LUAD.

## 2 Materials and methods

### 2.1 Data resource

The GSE26939, GSE31210, GSE19188, and GSE135222 were downloaded from the GEO database (https://ncbi.nlm.nih.gov/geo/). The immune-related profiles of LUAD were downloaded from the InnateDB database (https://www.innatedb.ca/) and Immort database (https://www.immport.org). Immunotherapy cohorts IMvigor210 and GSE93157 were included for analysis of immune therapy response (Bhattacharya et al., 2018; Breuer et al., 2013).

### 2.2 Acquisition of M2 macrophage-related genes

We analyzed immune-related genes using the Weighted Gene Co-expression Network Analysis (WGCNA), and then constructed the network by one-step method to obtain the module genes that were most related to M2 macrophage. The module genes that were most related to M2 macrophages were identified as M2 macrophage-related hub genes. Then, univariate Cox regression analysis was carried out to confirm M2 macrophage-related prognostic genes. Prognostic genes with p < 0.05 were finally enrolled.

### 2.3 Functional enrichment

Using the “clusterprofiler” package, Gene Ontology (GO) analysis was performed on prognostic feature genes, categorizing GO functions into three parts: Cellular Component (CC), Molecular Function (MF), and Biological Process (BP). Additionally, the Kyoto Encyclopedia of Genes and Genomes (KEGG) enrichment analysis was conducted, with significance set at p < 0.05 for enrichment.

### 2.4 Genotyping based on characteristic genes

The “ConsensusClusterPlus” package was used to conduct consistency cluster analysis. The overall slope of the curve shows the smallest decline when K is 2, leading to the classification of patients in GSE26939 into two molecular subtypes. Then, differentially expressed genes (DEG) between two subtypes were confirmed by the “limma” package. Those genes with adj.p < 0.05 and |logFC| > 1.5 were regarded as DEGs.

### 2.5 Construction of M2 macrophage-related prognostic signature

We developed a risk model based on M2 macrophage-related genes using the machine learning algorithm known as least absolute shrinkage and selection operator (LASSO) regression. The risk score model was constructed by the following formula:

Risk score=∑Coefi * Expr i

“Expr” was the expression value of signature genes in the model, and “Coef” was the regression coefficient. Then, patients were divided into high- and low-risk groups according to the optimal cutoff of risk score of all LAUD samples. Kaplan-Meier survival curves and area under curve (AUC) were used to verify the performance of the signature. Univariate and multivariate Cox were used to verify the performance of the prognostic signature.

### 2.6 Analyses of clinical characteristics, immune cells, and immunotherapy

To further explore the role of the risk signature in the immune microenvironment. Based on the core algorithm of CIBERSORT (CIBERSORT.R script analysis), we utilized the markers of 22 immune cell types provided by the CIBERSORTx website (https://cibersortx.stanford.edu/) to compute the immune infiltration between high- and low-risk groups. Moreover, ImmuneScore, StromalScore, and EstimateScore were analyzed by the “ESTIMATE” package.

### 2.7 Single-cell transcriptome database analysis

The single-cell transcriptome profile (GSE131907) downloaded from the Gene Expression Omnibus (GEO) database, including 15 lung cancer samples, was selected for subsequent analyses.

Firstly, quality control of single-cell profiles was done by Seurat (v4.1.0). The quality control standards were as follows: (1) Each gene was detected in more than 3 cells. (2) Features of each cell were between 500 and 6,000, with 1,000–20,000 counts. (3) The percentage of mitochondrial genes and erythrocytes gene expression was less than 20%. We use the “NormalizeData” function for normalization and the “FindVariableFeatures” function for identifying hypervariable genes, which were with 0.1–3 average expression value and more than 0.5 dispersion. Batch correction between samples was performed by the “harmony” package. Principal component analysis (PCA) was used for dimensionality reduction, and the first 50 principal components were selected for downstream analysis. The *t*-distributed stochastic neighbor embedding (tSNE) algorithm was used for visualization.

The top 50 principal components with 0.2 resolution were used to identify subpopulations of tumor cells. The “FindAllMarkers” function was used to identify feature genes, and each model contained 10 genes. Cellscore was calculated by the “AddModuleScore” algorithm. The malignant epithelial cells were divided into high- and low-groups according to the middle value of Cellscore. The “Monocle2” package was used to analyze the single trajectory.

### 2.8 Statistical analyses

All the above statistical analysis was computed by R software (version 4.2.1, https://www.r-project.org/). P-value < 0.05 (two-sided) was used as the statistically significant threshold. The survival difference between the two groups was analyzed by Kaplan‒Meier analysis. Other statistical methods and algorithms used in this article are described in the corresponding steps.

## 3 Results

### 3.1 Screening of macrophage subtypes in LUAD

**FIGURE 1:**
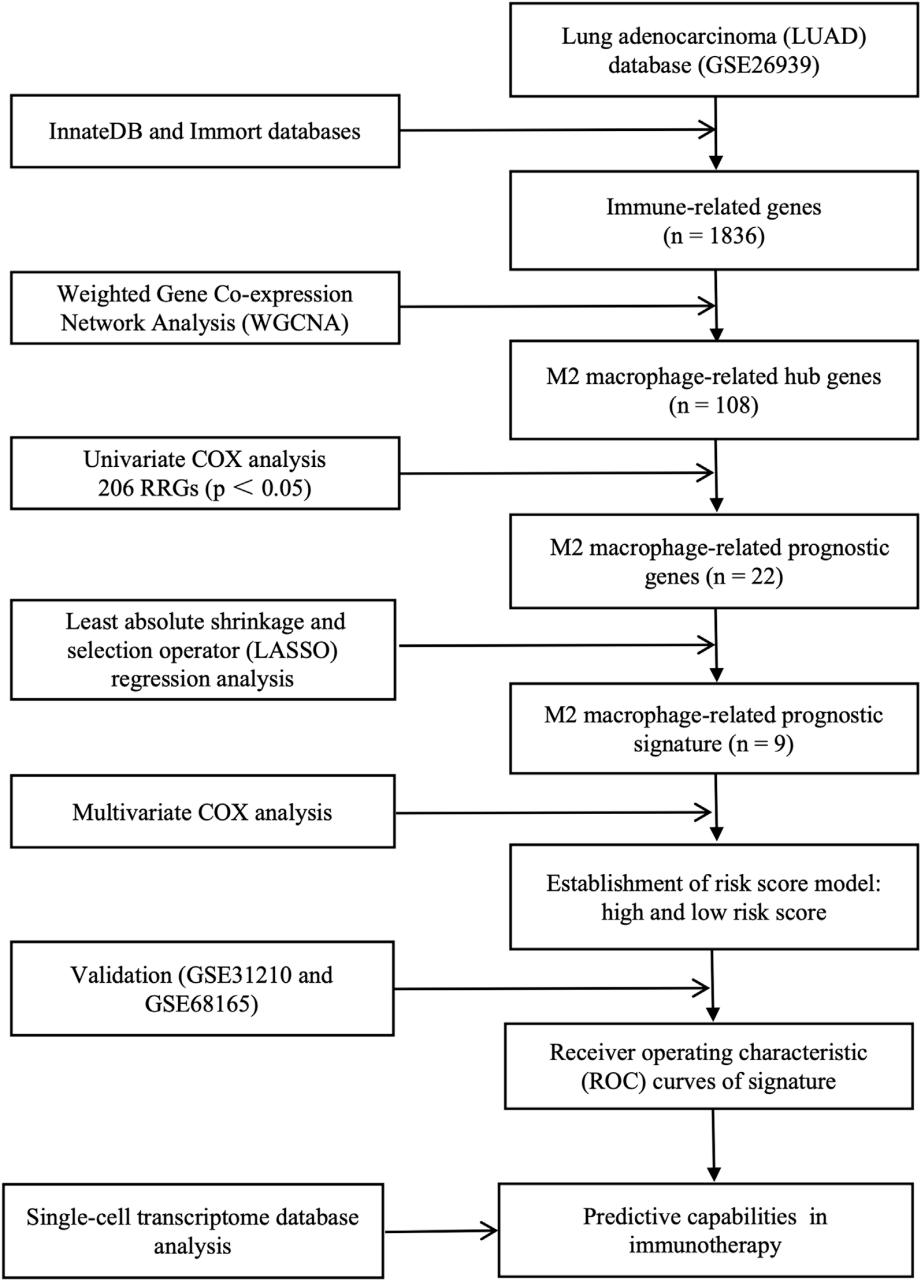
Complete workflow of our research. “n” denotes sample size. “p < 0.05” denotes the statistically significant threshold.

**FIGURE 2:**
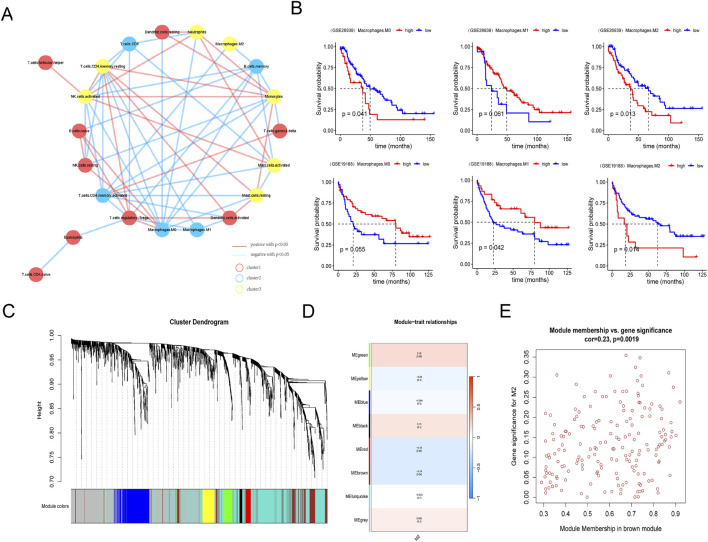
Screening of macrophage subtypes in LUAD. **(A)** Network diagram of infiltrating immune cells in lung adenocarcinoma samples. **(B)** Comparison of survival between high or low M0, M1, and M2 macrophage in GSE26939 (upper column) and GSE19188 databases (lower column). **(C)** Hierarchical clustering tree view by weighted gene co-expression network analysis. **(D)** Heat map of module phenotypic correlation. **(E)** Internal gene scatter map in brown module.

The workflow of the study is shown in Figure 1. Immune cells were divided into three different clusters, and we evaluated the correlation of various immune cells using correlation analysis (Figure 2A). Macrophages are a significant constituent part of TME. To confirm the relationships between macrophages and survival in patients with LUAD, patients were divided into high- and low-macrophage groups based on macrophage infiltration level. Survival analysis suggests that patients in the high M2 group have a worse prognosis, while those in the high M1 group have a better prognosis (Figure 2B). Therefore, the M2 macrophage was chosen for further exploration.

### 3.2 Screening of M2 macrophage-related hub genes

Then, WGCNA was used to identify M2 macrophage-related genes in LUAD. Using the InnateDB and Immort databases, 1836 immune-related genes were obtained from the GSE26939 database (Figure 2C). Seven modules were identified by WGCNA, and the brown module was significantly associated with M2 macrophage (Figure 2D). Thus, 108 hub genes in the brown module were selected for further analysis (Figure 2E; Supplementary Table S1).

### 3.3 Screening for M2 macrophage-related prognostic genes

**FIGURE 3:**
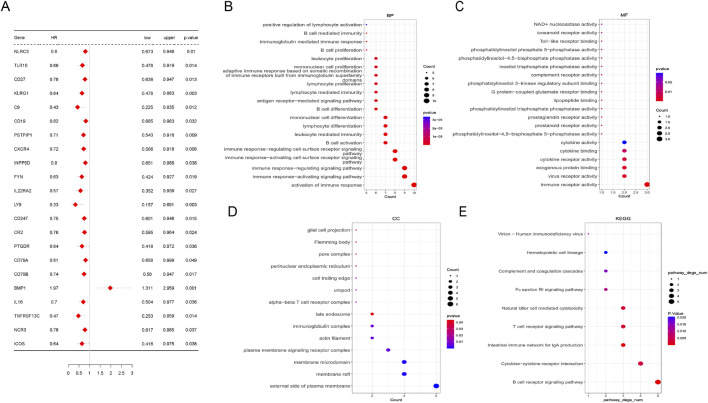
Screening of M2 macrophage-related hub genes. **(A)** Univariate Cox analysis of 22 prognostic genes in lung adenocarcinoma. **(B–E)** Pathways enriched by Gene Ontology (GO) analysis and Kyoto Encyclopedia of Genes and Genomes (KEGG) analyses based on 22 prognostic genes. Biological process (BP), molecular function (MF), and cellular component.

To address the critical genes involved in the biological function of M2 macrophage, univariate Cox regression analysis was conducted. Twenty-two genes among 108 hub genes associated with the prognosis of LUAD were confirmed by univariate analysis. Except for BMP1 (bone morphogenetic protein 1), the remaining 21 genes were considered favorable factors in LUAD (Figure 3A). GO (Gene Ontology) analyses showed that the above 22 prognostic genes were significantly enriched in the activation of the immune response, immune response−activating signaling pathway, immune receptor activity, etc (Figures 3B–D). Similarly, Immune-related pathways, like B cell and T cell receptor signaling pathways, were significantly enriched in KEGG (Kyoto Encyclopedia of Genes and Genomes) analyses (Figure 3E).

### 3.4 Molecular subtypes of LUAD

**FIGURE 4:**
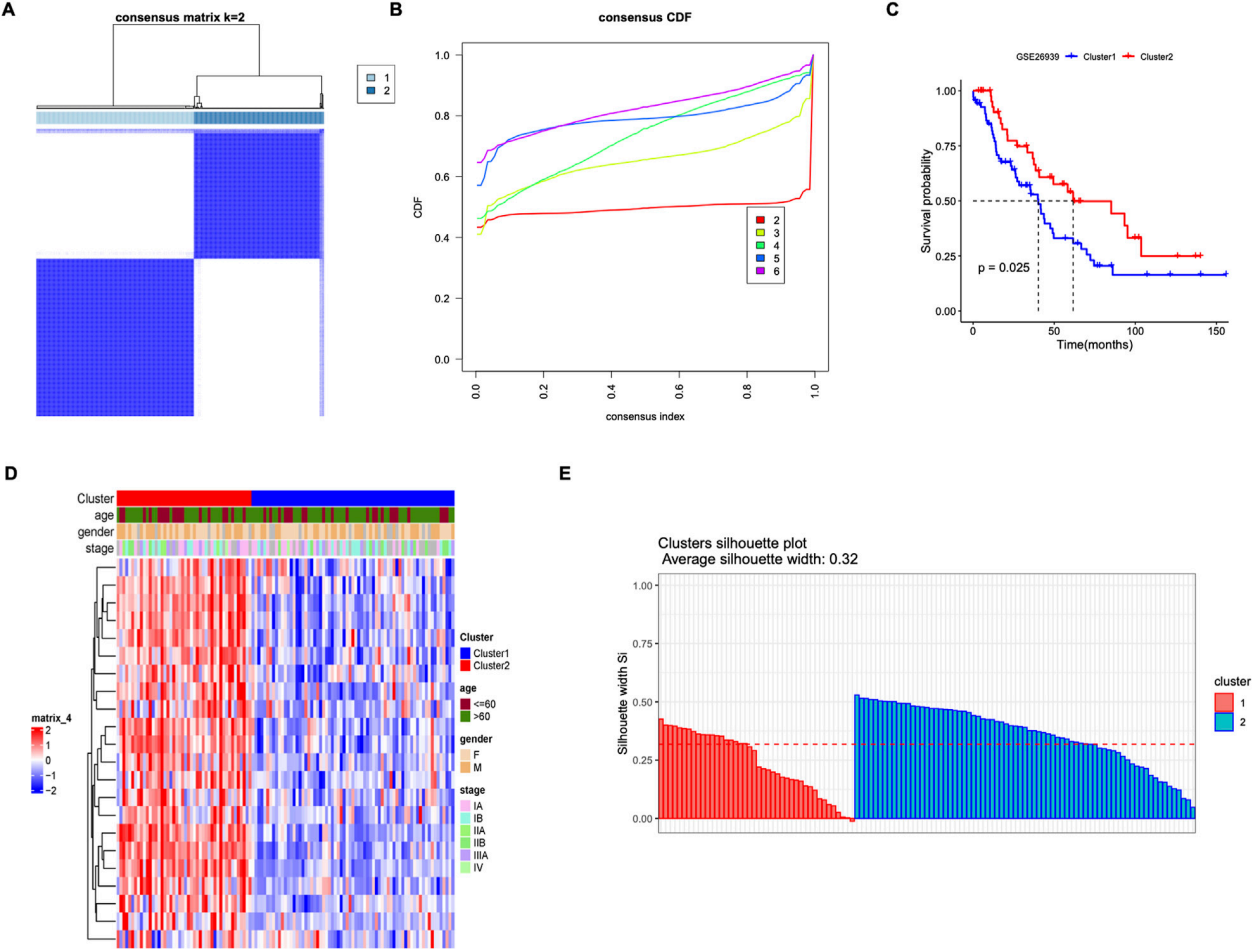
Molecular subtypes based on M2 macrophage-related prognostic genes. **(A)** Matrix heat map when k = 2. **(B)** Consistent cumulative distribution function (CDF) diagram, which shows the cumulative distribution function when k takes different values. **(C)** Survival curve between subtypes in GSE26939. **(D)** Heat map of correlation between subtypes and clinical features. **(E)** Profile of subtype correctness.

As we know, LUAD is full of heterogeneity. To better identify the different populations, patients with LUAD were classified into two molecular subtypes in the GSE26939 database by consistent cluster analysis (Figures 4A, B). There was a significant difference in survival outcomes between the two clusters (p = 0.0025) (Figure 4C), with different gene expressions, clinic characteristics, and profile of subtype correctness (Figures 4D, E).

**FIGURE 5:**
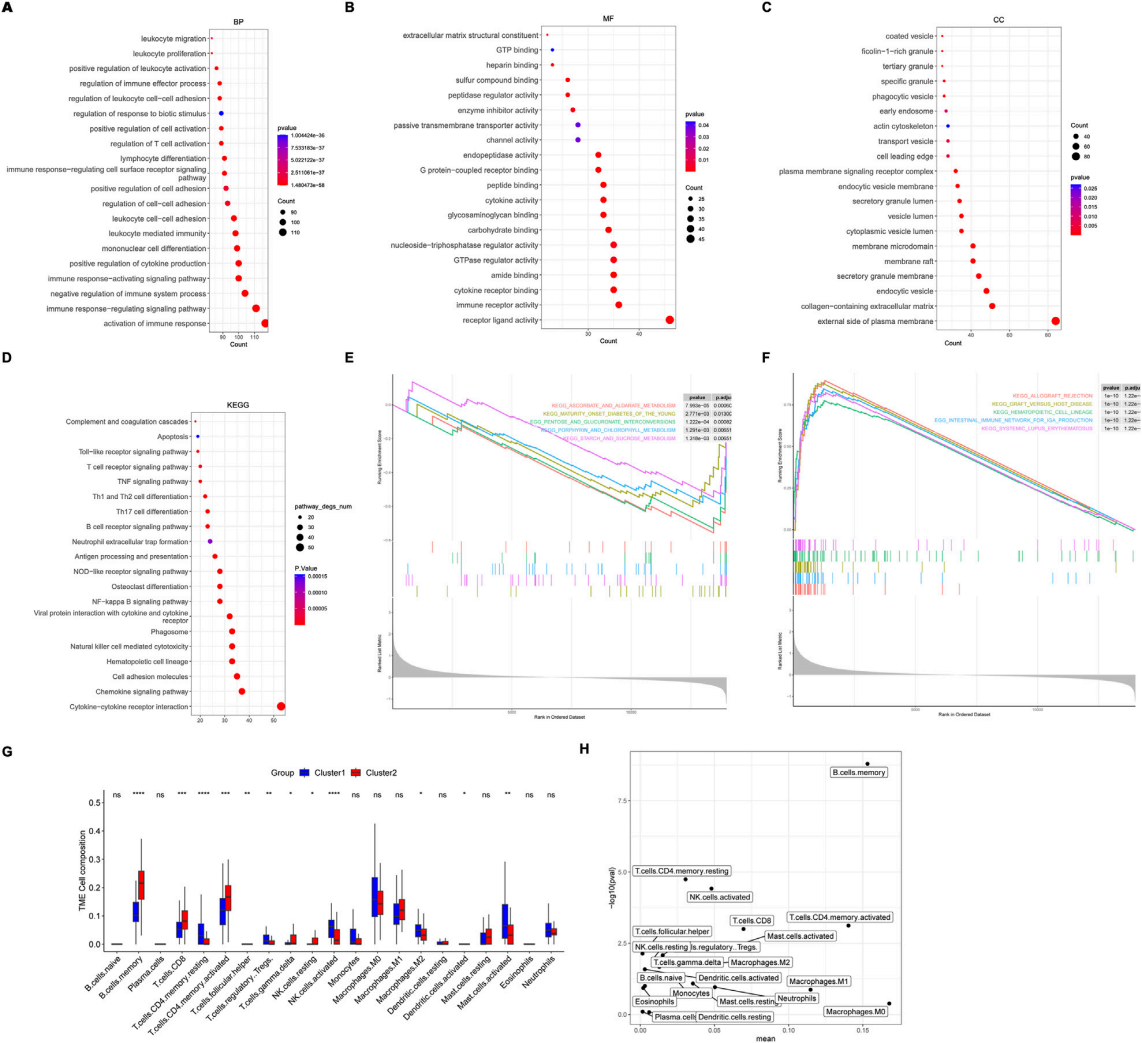
Pathways enrichment and immune cells between molecular subtypes. **(A–D)** The top 20 pathways enriched by Gene Ontology (GO) analysis and Kyoto Encyclopedia of Genes and Genomes (KEGG) analysis based on differentially expressed genes (DEGs) between molecular subtypes. Biological process (BP), molecular function (MF), and cellular component. **(E)** The top 5 inhibition and activation **(F)** pathways enriched by Gene Set Enrichment Analysis (GSEA). **(G, H)** Different expression of immune cells between molecular subtypes.

Then, differentially expressed genes (DEGs) between these two clusters were analyzed. There were 772 DEGs identified, with 75 down-regulated DEGs and 697 up-regulated DEGs. GO and KEGG analysis revealed that activation of immune response and interaction of cytokine-cytokine receptors were significantly enriched (Figures 5A–D). The top 5 inhibition and activation pathways were also shown by Gene Set Enrichment Analysis (GSEA) (Figures 5E, F).

Interestingly, immune cells, including M2 macrophage, resting CD4 memory T cells, regulatory T cells, activated NK cells, and mast cells, were lower in cluster 2, indicating a favorable prognosis (Figures 5G, H).

### 3.5 Construction of an M2 macrophage-related prognostic signature

**FIGURE 6:**
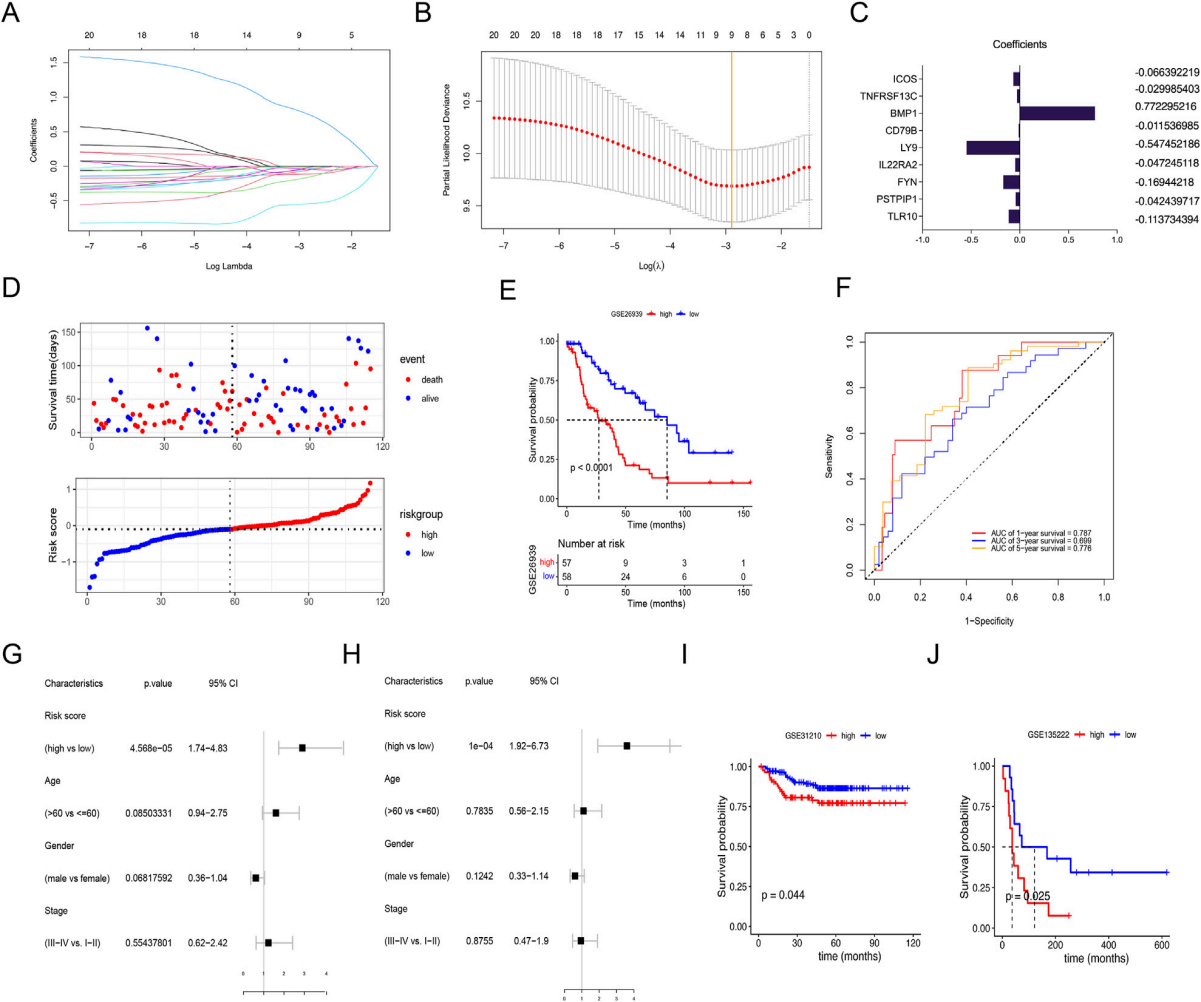
Using lasso regression analysis to construct the risk signature. **(A)** Lasso coefficient distribution of 22 M2 macrophage-related prognostic genes. **(B)** Tuning parameter (λ) selection cross-validation error curve. Vertical lines were drawn at the optimal values. **(C)** Regression coefficient corresponding to the 9 M2 macrophage-related prognostic genes screened. A larger absolute value of the coefficient represents a higher correlation. **(D)** Survival status of patients with high- and low-risk scores. **(E)** Survival curves for LUADs with high- and low-risk scores. **(F)** Receiver operating characteristic (ROC) curves for 1-, 3- and 5-year overall survival in LUAD cohort. **(G)** Univariate and multivariate **(H)** analyses of clinical features and risk signature. **(I, J)** Validation of the risk signature in the database of GSE31210 and GSE68165 with high- or low-risk scores.

To explore a simple and reliable therapy strategy, a risk prognostic model was constructed based on the 22 M2 macrophage-related prognostic genes. Nine genes were confirmed by LASSO regression analysis in GSE26939 (Figures 6A, B). The coefficient of each gene in the model is shown in Figure 6C. We divided the patients into high-risk and low-risk groups based on the median risk score (Figure 6D). Patients with LUAD in the low-risk group had longer overall survival (OS) than in the high-risk group (p < 0.0001) (Figure 6E). ROC curves were plotted to estimate the performance of the risk model. The AUC value of ROC curves at 1, 3, and 5 years was 0.787, 0.699, and 0.776, respectively, indicating this signature scoring system had a good predictive performance (Figure 6F). The univariate and multivariate analyses showed that the signature based on risk score was an independent prognostic factor in LUAD (Figures 6G, H). Moreover, the verification gene sets (GSE31210 and GSE68165) further demonstrated that the patients with the low-risk had superior survival than the high-risk group (Figures 6I, J).

**FIGURE 7:**
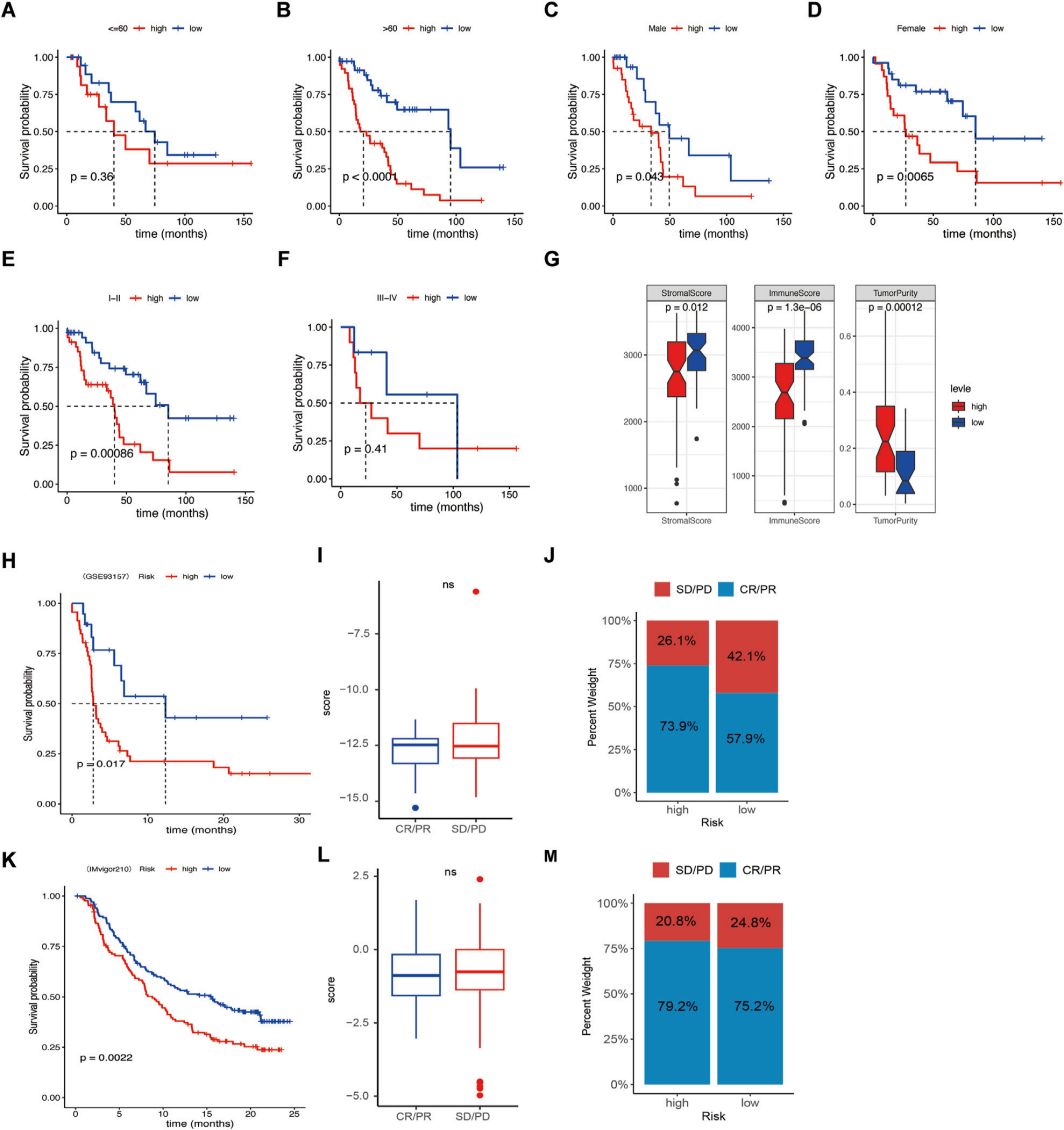
Relationships between risk score and clinical features and immune response. **(A–F)** Survival difference between age, sex, and clinical stage of the high- or low-risk score groups. **(G)** Correlation analysis between risk score and stromal score, immune score, and tumor Purity. **(H, K)** Kaplan–Meier curves of overall survival time of the high- and low-risk score groups in the metastatic non-small cell lung cancer (NSCLC) sample and in the metastatic urothelial carcinoma (mUC) sample. **(I, L)** Correlations of response (complete response/partial response) and nonresponse (stable disease/progressive disease) to immunotherapy in different risk score groups. **(J, M)** Relative percent of response and nonresponse to immunotherapy in the high- or low-risk score groups in the metastatic NSCLC sample and the mUC sample. Not significant.

Further subgroup analysis suggested that the low-risk group aged over 60 years old and stage Ⅰ-Ⅱ had longer survival than the high-risk group, regardless of sex in GSE26939 (Figures 7A–F). No significant survival differences were found regarding tumor stage Ⅲ-Ⅳ, age of ≤60.

### 3.6 Relationships between risk signature and immunotherapy response

Since infiltrating immune cells varies in the different molecular subtypes and risk score groups, we wonder if the signature was associated with immunotherapy response. Our data revealed that the high-risk group had lower stromal score and immune score, but higher Tumor Purity, indicating patients with low-risk group had better effects of immunotherapy (Figure 7G). Then, the GSE93157 database, including NSCLC patients receiving PD1-targeting antibodies pembrolizumab or nivolumab, and the IMvigor210 database, including metastatic urothelial carcinoma patients receiving PD-L1-targeting antibodies atezolizumab were used. The results showed that the patients with low risk had better immunotherapy efficacy compared to the high-risk group (Figures 7H, K). While the risk was not associated with the response rate (CR/PR) and nonresponse rate (SD/PD) (Figures 7I, J, L–M). Taken together, our data suggest that the signature was a potential biomarker for NSCLC patients receiving immunotherapy.

### 3.7 Single-cell transcriptome database analysis

**FIGURE 8:**
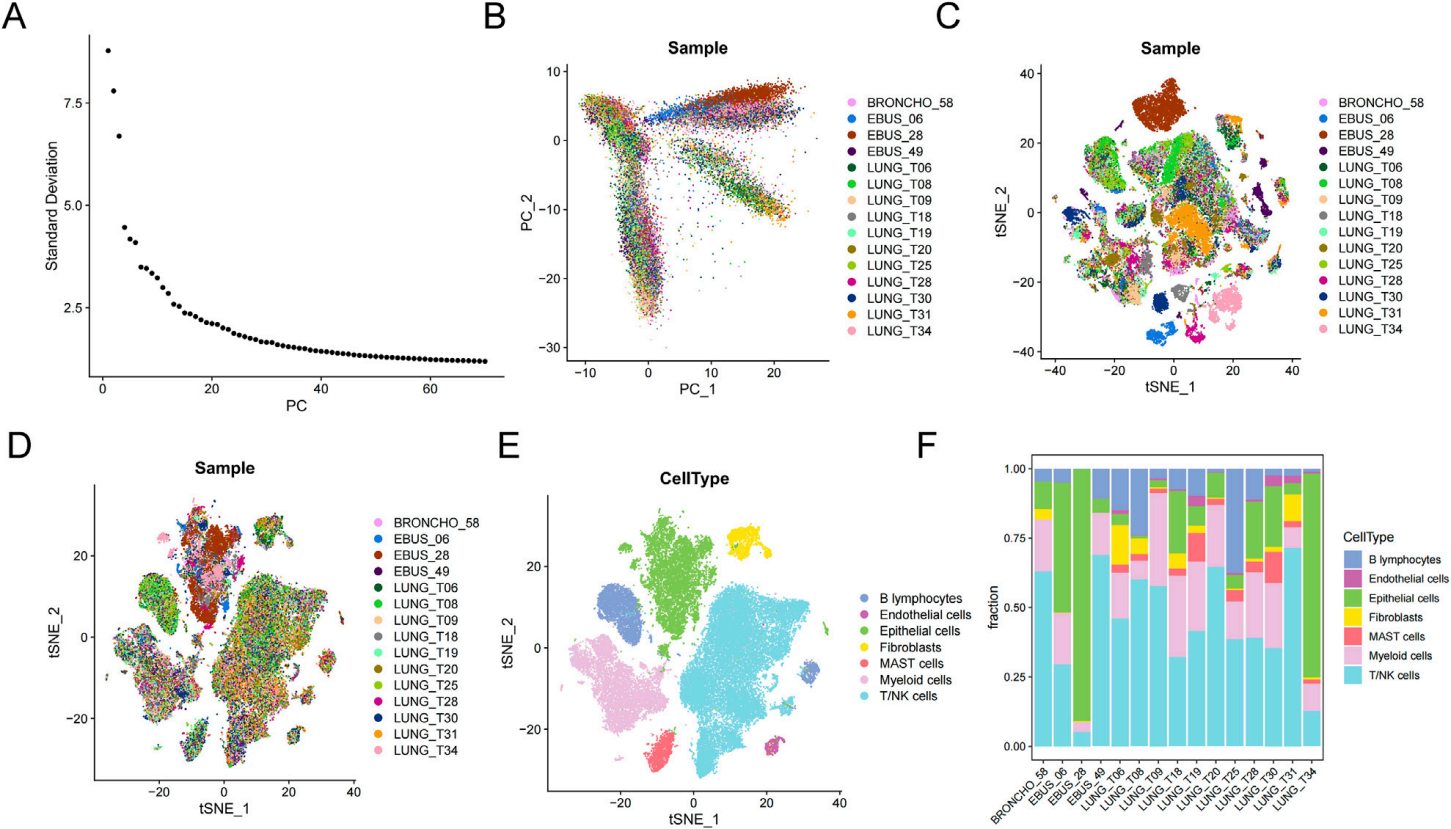
Microenvironment cell landscape. **(A)** ElbowPlot of principal component analysis (PCA). **(B)** PCA of 15 lung cancer samples. **(C)** Sample cell distribution before de-batch effect. **(D)** Sample cell distribution after de-batch effect. **(E)** t-Distributed Stochastic Neighbor Embedding (tSNE) distribution of different cell types. **(F)** Percentage of cell types.

Fifteen primary lung cancer samples in the single-cell transcriptome profiles (GSE131907) were selected for analysis. After quality control, 27,578 genes within 51,935 cells were obtained. PCA (principle component analysis) results showed significant batch effects among samples (Figures 8A, B). After using Harmony to remove batch effects between samples (Figures 8C, D), UMAP (Uniform Manifold Approximation and Projection) showed seven major cell types, composed of B lymphocytes, endothelial cells, epithelial cells, fibroblasts, MAST cells, myeloid cells, and T/NK cells (Figure 8E). The proportion of cells in each sample was heterogeneous (Figure 8F).

**FIGURE 9:**
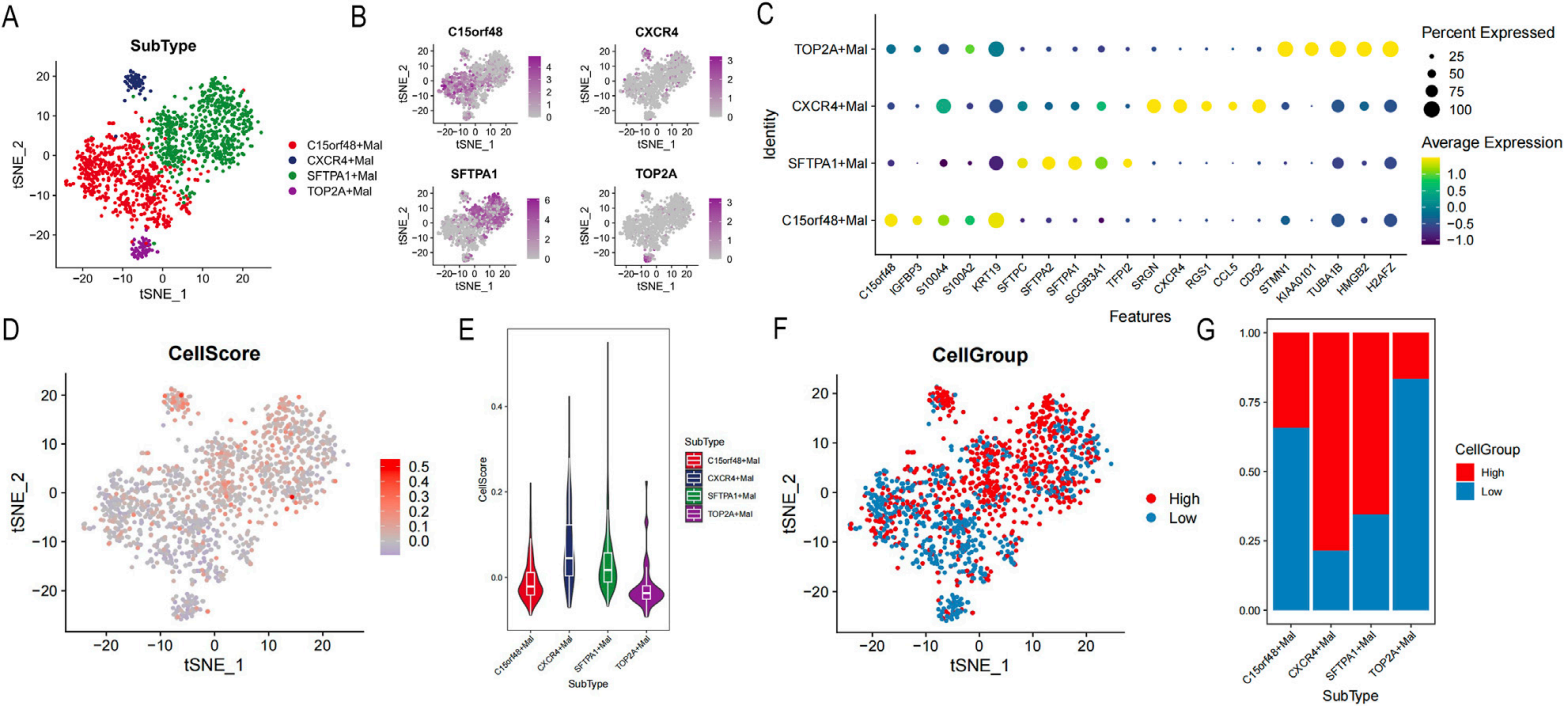
Identification of tumor cell subsets. **(A)** Distribution of each cell subgroup. **(B)** t-Distributed Stochastic Neighbor Embedding (tSNE) map of highly expressed genes. **(C)** Bubble map of the top 5 characteristic gene expressions in each subgroup. **(D)** tSNE map of cellscore. **(E)** Violin distribution map of different cell subsets of cellscore. **(F)** tSNE map of high or low cellgroup. **(G)** The proportion of high or low cellgroup in different subgroups.

In this study, four different lung cancer subtypes were identified were defined: sftpa1+mal, c15orf48+mal, cxcr4+mal, and top2a + mal, according to the high expression genes of each subtype (Figures 9A, B). Based on FindAllMarkers, the top 5 characteristic genes of each subtype were identified (Figure 9C). Sftpa1+mal over-expressed sftpa1, sftpa2, sftpc, and other genes, and these genes were significantly enriched in biological processes such as MHC complex assembly, antigen treatment, and presentation by GO and KEEP analysis (Supplementary Figure S1). C15orf48+mal highly expressed c15orf48, IGFBP3, S100A4, and other genes, which were significantly enriched in the regulation of cell morphogenesis, cell-matrix adhesion, and other biological processes (Supplementary Figure S2). Cxcr4+mal highly expressed SRGN, CXCR4, CD52, and other genes, which were significantly enriched in the regulation of T cell activation, T cell receptor signaling pathway, and lymphocyte differentiation (Supplementary Figure S3). Top2a + mal highly expressed cell cycle marker TOP2A, significantly enriched in the cell cycle, nucleus division, and DNA replication, suggesting that the tumor was in an active cell proliferation state (Supplementary Figure S4).

To further distinguish lung cancer lineages at the single-cell level, lung cancer was divided into high- and low-cell groups according to cell scores (Figure 9D). Our data revealed that subtypes of sftpa1+mal and cxcr4+mal had higher cell scores, suggesting those two subtypes had more malignant character (Figure 9E). According to cell scores, malignant epithelial cells were divided into high- and low-cell groups (Figures 9F, G). In addition, DEGs of the high- and low-cell groups indicated that DEGs were significantly enriched in tumor immune-related processes, such as regulation of peptidase activity, humoral immune response, assembly of MHC class Ⅱ protein complexes, antigen processing, and presentation (Supplementary Figure S5).

**FIGURE 10:**
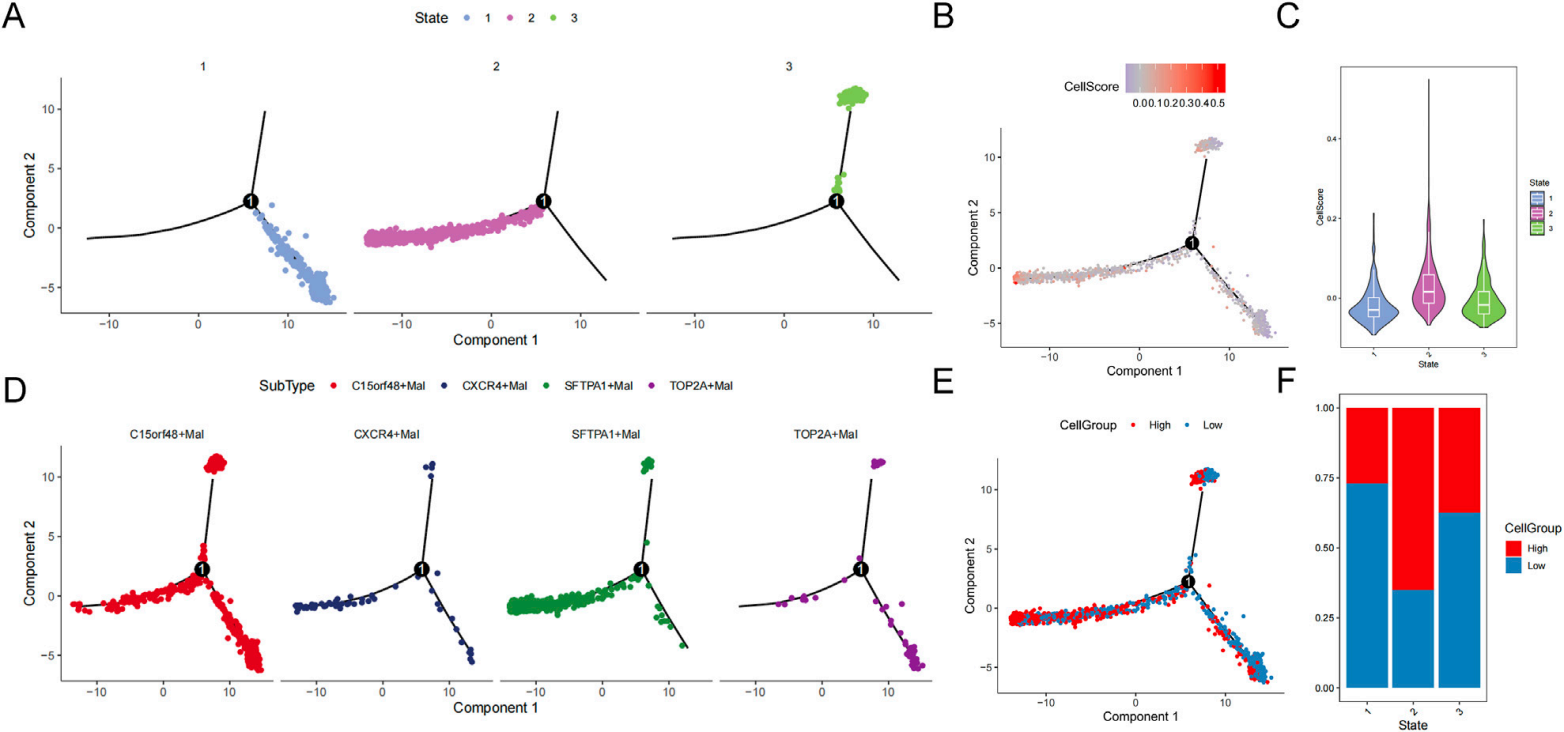
Trajectory analysis of malignant epithelial cells. **(A)** Trajectory distribution of state. **(B)** Trajectory distribution of cellscore. **(C)** Violin distribution of cellscore in different states. **(D)** Trajectory distribution of subsets. **(E)** Trajectory distribution of cellgroup. **(F)** Proportion of cellgroup in different state.

Then developmental trajectories were constructed, and three differentiation states were obtained (Figures 10A–C). The developmental trajectory of subtypes in state 1 to state 3 was relatively uniform. In the state 1 to state 2 developmental trajectories, the c15orf48+mal subtype was in the early or middle stage of cell differentiation, and the sftpa1+mal subtype was in the late stage of cell differentiation (Figure 10D). In these two developmental trajectories, the cell scores and the high-cell groups were increased, suggesting the malignant degree is rising (Figures 10E, F).

## 4 Discussion

Over the years, immunotherapy has significantly improved survival in LUAD without driver genes. PD-L1 expression is a currently recognized and strongly recommended tumor marker (Dora et al., 2023; Doroshow et al., 2021; Sanchez-Magraner et al., 2023), however, it is an imperfect biomarker. Other biomarkers, such as neoantigens, genetic, epigenetic signatures, microbiome composition, and factors in TME, are also used to predict immunotherapy response and prognosis in LUAD (Mino-Kenudson et al., 2022). ICIs aim to enhance the anti-tumor effect by activating effector T cells in TME, which involves immune escape and tumor progression by immunosuppressive cells and molecules (Binnewies et al., 2018; Cristescu et al., 2018). However, biomarkers are lacking to predict the efficacy of ICIs in clinical practice in TME. Macrophages are the most common immune cells in TME. Our study demonstrated that M2 macrophages were an unfavorable factor for patients with LUAD, and the signature based on M2 macrophages was a promising biomarker to predict the survival and immunotherapy response in LUAD. Single-cell transcriptome analysis is a useful tool to predict molecular heterogeneity and give a highlight to a more precise classification of lung cancer.

Other macrophage-related prognostic models to guide the management of LUAD were also reported (Li et al., 2023; Xiang et al., 2024; Zhu et al., 2022). Li et al. constructed a macrophage-related index for predicting prognosis and immunotherapy response based on 22 genes using 10 machine-learning algorithms (Li et al., 2023). Xiang et al. built a novel gene signature of 12 differentially expressed genes and identified TRIM28 as a potential biomarker for treatmenting LUAD (Xiang et al., 2024). In contrast to those studies, we used less macrophage-related genes and had higher AUC values of ROC curves at 1 and 5 years. Besides, we also provide single-cell transcriptome analysis to address the molecular subtypes and heterogeneity in LUAD, which could be helpful in risk stratification. Of note, the genes were diverse between these three studies, as the database and research methods were different. Moreover, key genes in the tumor microenvironment affecting the development and immunotherapy efficacy of LUAD need further validation.

M1/M2 polarization is dynamic to adapt tumor progression (Yang and Zhang, 2017). Emerging reports have shown a positive correlation between macrophage density and poor survival (Festekdjian and Bonavida, 2024). Consistent with the reports, we found that patients with high-M2 macrophage had worse prognosis compared to those patients with low-M2 macrophage. The underlying mechanisms lie in that cancer cells can secrete cytokines, such as IL10, IL12, IL 6, and TNF, facilitating M2-like polarization, then exerting immunosuppressive effects, and finally accelerating cancer progression (Sarode et al., 2020). In lung cancer, transforming growth factor beta (TGF-β), IL-10, cytokines, and chemokines released by M2 macrophages can promote tumor growth and infiltration (Wang et al., 2019; Yang et al., 2019). In addition, M2 macrophages (CD163+) could promote angiogenesis by releasing angiogenic growth factors such as vascular endothelial growth factor A (VEGF-A) and VEGF-C in NSCLC (Hwang et al., 2020). However, LUAD has great heterogeneity, especially in patients with different driver genes, which may affect the roles of macrophages. Therefore, more research is needed to explore the potential mechanisms and clinical implications.

Nine macrophage-related prognostic genes (TLR10, PSTPIP1, FYN, IL22RA2, LY9, CD79B, BMP1, TNFRSF13C, ICOS) were screened for construction of prognostic signature in LUAD. Nishikawa S et al. found phosphorylated FYN expression was associated with poor relapse-free survival and overall survival in patients with LUAD after lung resection (Nishikawa et al., 2019). In line with FYN, LUAD patients with high expression of TNFRSF13C (BAFFR) had worse survival (Dimitrakopoulos et al., 2019). Rochigneux P reported that inducible T-cell co-stimulator (ICOS)^+^ CD4^+^ T cells were closely associated with better survival for patients receiving pembrolizumab in NSCLC (Rochigneux et al., 2022). Moreover, Wu G et al. suggested that ICOS was closely correlated with poor outcomes in multiple cancers, especially LUAD, and was a good biomarker of OS in LUAD (Wu et al., 2022). Our data suggested that BMP1 plays the opposite role compared to the other eight genes in the prognostic signature. X Wu reported that downregulation of BMP1 leads to suppression of TGFβ and matrix metalloproteinases 2 (MMP2) and MMP9, and finally decreased tumor invasion in NSCLC (Wu et al., 2014). In addition, different BMP1 isoforms may impact NSCLC disease progression (Donovan et al., 2023), however, insights into the mechanisms remain unclear.

The association between DEGs and M2 macrophages is worthy of attention and research because some of these macrophage-related prognostic genes were significantly related to immune response. IL-22RA2 was closely associated with macrophage effector mechanisms in experimental neuroinflammation (Beyeen et al., 2010). ICOS-mediated ICOS ligand triggering modulates the activity of human M1 and M2 cells, eliciting an overall anti-inflammatory effect (Gigliotti et al., 2023). Interestingly, the cytoskeletal adaptor PSTPIP1 controls extracellular matrix degradation and filopodia formation in macrophages, suggesting a potential target for therapeutic strategy in autoinflammatory disease (Ishiguro et al., 2020). The CD79b-directed antibody drug conjugate (ADC) polatuzumab vedotin targets macrophages in follicular lymphoma (Gordon et al., 2023). Altogether, more basic research and translational clinical studies are needed to confirm the above studies.

ICIs have demonstrated improved OS compared with chemotherapy in non-oncogene-addicted metastatic NSCLC (Hendriks et al., 2023), while immunotherapy biomarkers are lacking. Our study revealed that the signature based on M2 macrophage-related prognostic genes was a potential biomarker for NSCLC patients receiving immunotherapy. Our study found that patients with high risk tended to have a “cold” tumor phenotype, with a lower proportion of activated T cells and a higher proportion of M2 macrophage, indicating poor response to immunotherapy. Thus, integral evaluation of Tumor microenvironment, including M2 macrophage and PD-L1, is essential before immunotherapy in lung cancer. Of note, Mechanical studies are also necessary. M2 macrophages could release immunosuppressive cytokines in tumors to weaken the function of T cells, leading to an immunosuppressive TME (Bui and Bonavida, 2024). However, the relationships between the efficacy of ICIs and different subtypes of M2 macrophages were unclear. Yamaguchi, Y et al. reported that PD-L1 blockade could restore CAR T cell activity through IFN-gamma-regulation of CD163+ M2 macrophages, suggesting the potential value of the combination of CAR T cells and ICIs in solid tumors to enhance therapeutic efficacy (Yamaguchi et al., 2022). Besides, the interaction and mechanism between PD-L1 expression and M2 macrophages are worthy of further study, which could provide a promising strategy in cancer immunotherapy (Zhao et al., 2024). More importantly, the signature needs to be confirmed in multicenter clinical trials.

Single-cell sequencing analysis is being more and more used in exploring the heterogeneity of tumor cells in TME. Lung cancer is a solid tumor originating from malignant epithelial cells. So, we constructed a prognostic model, aiming to analyze the model at the single-cell level. The malignant epithelial cells were divided into high- and low-cell groups based on the model score, and the pseudo temporal analysis showed that the cell subgroups with high scores were located at the end of the differentiation trajectory. The higher degree of the epithelial malignancy cells in the late stage of differentiation, the worse the prognosis of the LUAD patient may be, which coincides with the model score. Besides, our data found that subtypes of sftpa1+mal and cxcr4+mal in LUAD were with worse biological behavior. Of note, the result was different in other studies. Sorin M et al. reported that TAM was the most common cell in LUAD patients, accounting for 34.1% of immune cells, and CD163+ TAM (M2-like) was the most invasive structure (Sorin et al., 2023). Thus, basic, and translational research were wanted in the future.

This study has some limitations worth mentioning. Firstly, *in vivo*, and *in vitro* validation were lacking to explore the underlying mechanisms of immune efficacy affected by M2 macrophage-related prognostic genes in LUAD. And the key gene involved in regulating the immunotherapy response should also be addressed. Secondly, relationships between driving genes (EGFR and ALK) and M2 macrophage-associated immune response in LUAD were not further analyzed. Last, the clinical significance of different lung cancer subtypes of single cell sequencing in managing immunotherapy remains explored.

## 5 Conclusion

In summary, M2 macrophages were significantly associated with worse survival in LUAD. A risk signature based on M2 macrophage-related genes was a promising independent prognostic factor for patients with LUAD. More importantly, the signature was a potential biomarker for NSCLC patients receiving immunotherapy. Single-cell transcriptome analysis was a valuable tool for defining molecular subtypes and malignant degree. In the further, the necessity for more extensive translational research on M2 macrophage or M2 macrophage-related genes was needed to enable individual therapies for patients with LUAD.

## Data Availability

The original contributions presented in the study are publicly available. This data can be found here: [https://ncbi.nlm.nih.gov/geo/GSE26939, GSE31210, GSE19188 and GSE135222], [https://www.innatedb.ca/IMvigor210], and [https://www.immport.org/GSE93157].
