# Supplementary figures and images for: Integrated analysis of M2 macrophage-related gene prognostic model and single-cell sequence to predict immunotherapy response in lung adenocarcinoma
Source: Front Genet. 2025 Feb 3;16:1519677. doi: 10.3389/fgene.2025.1519677 (PMC11830816; doi:10.3389/fgene.2025.1519677)

KEGG Pathway

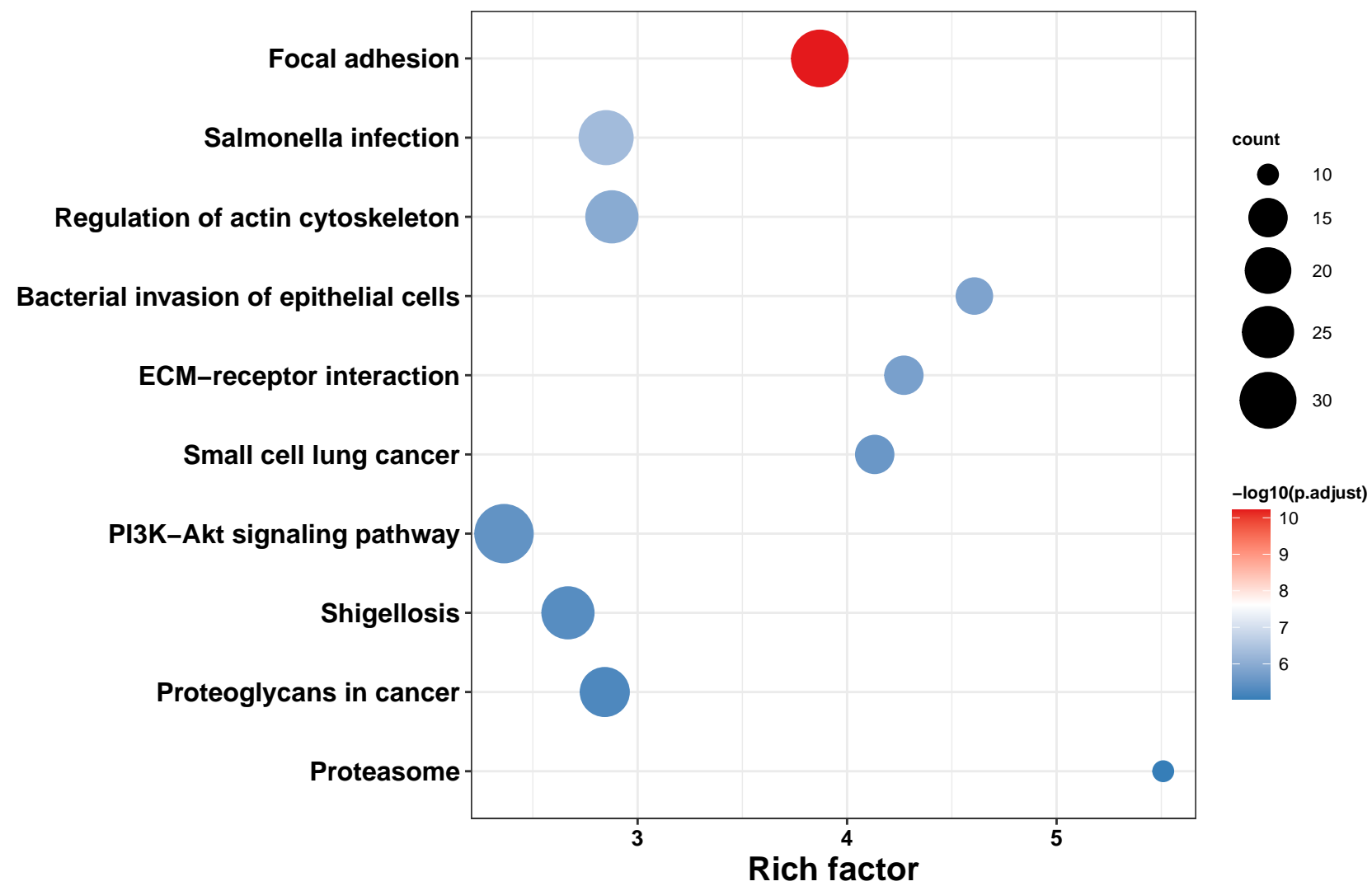

GO Biological Process

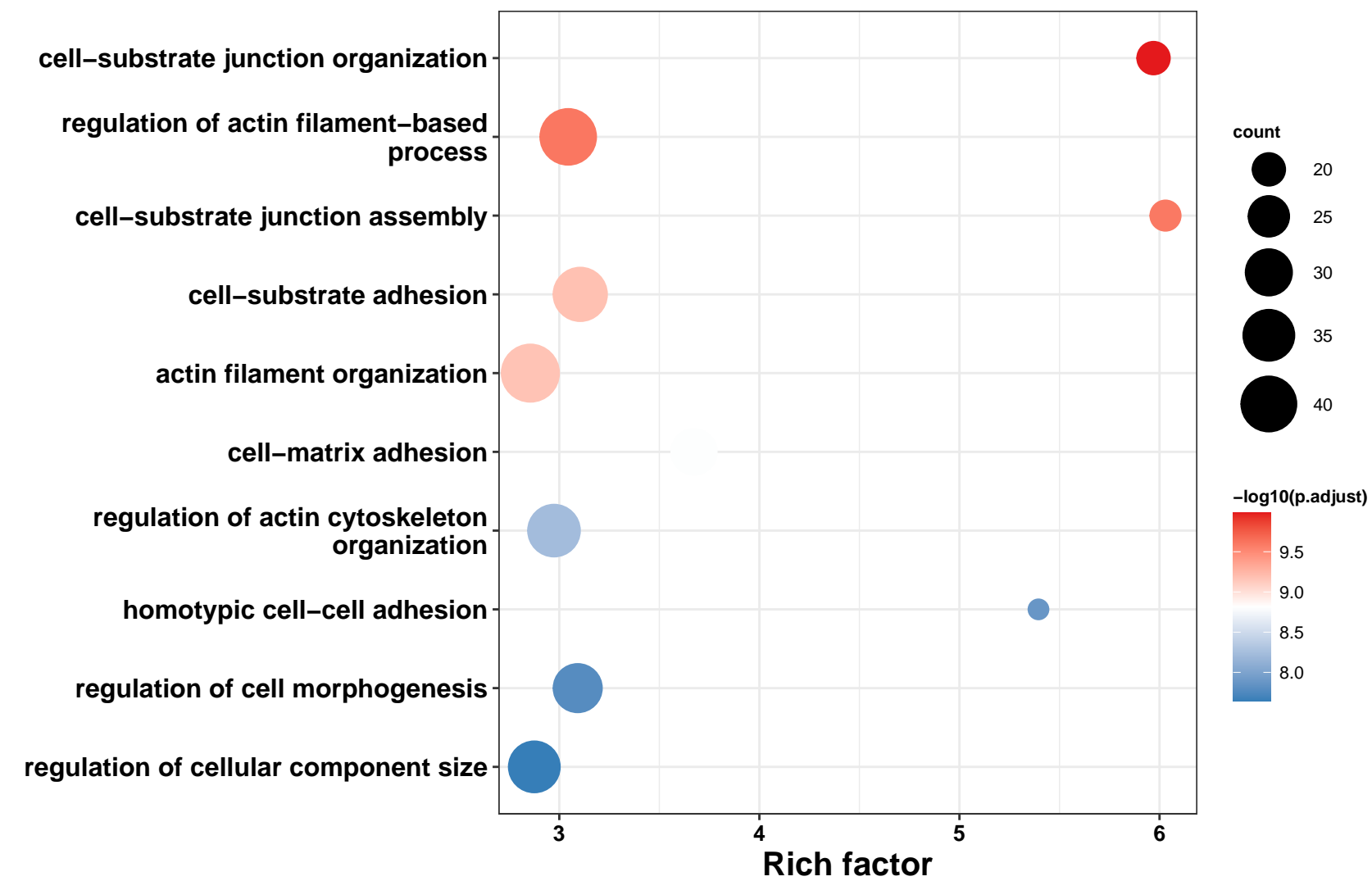

GO Molecular Function

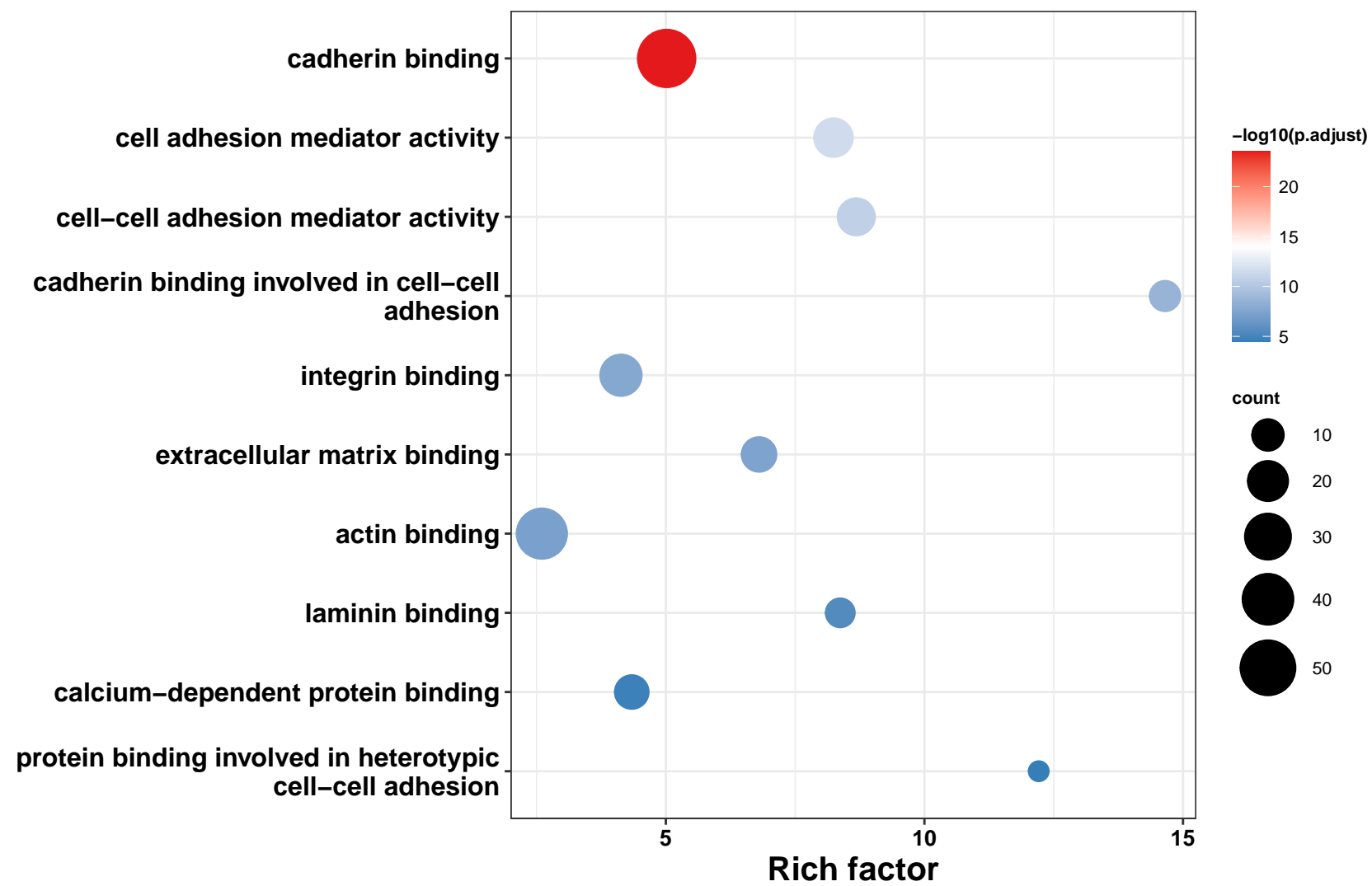

GO Cellular Component

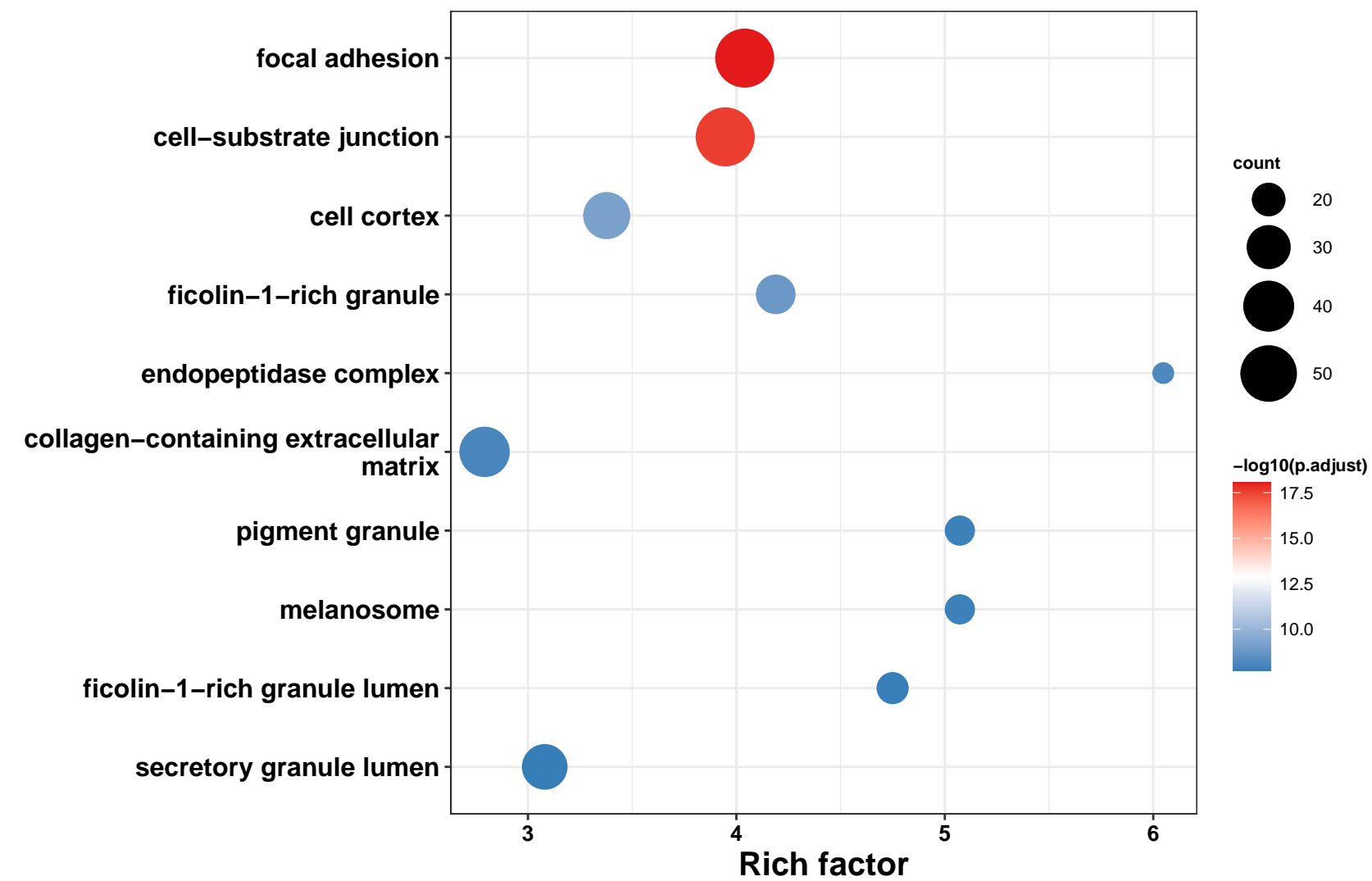

Supplement: Supplementary file 1 [file DataSheet2.pdf]

KEGG Pathway

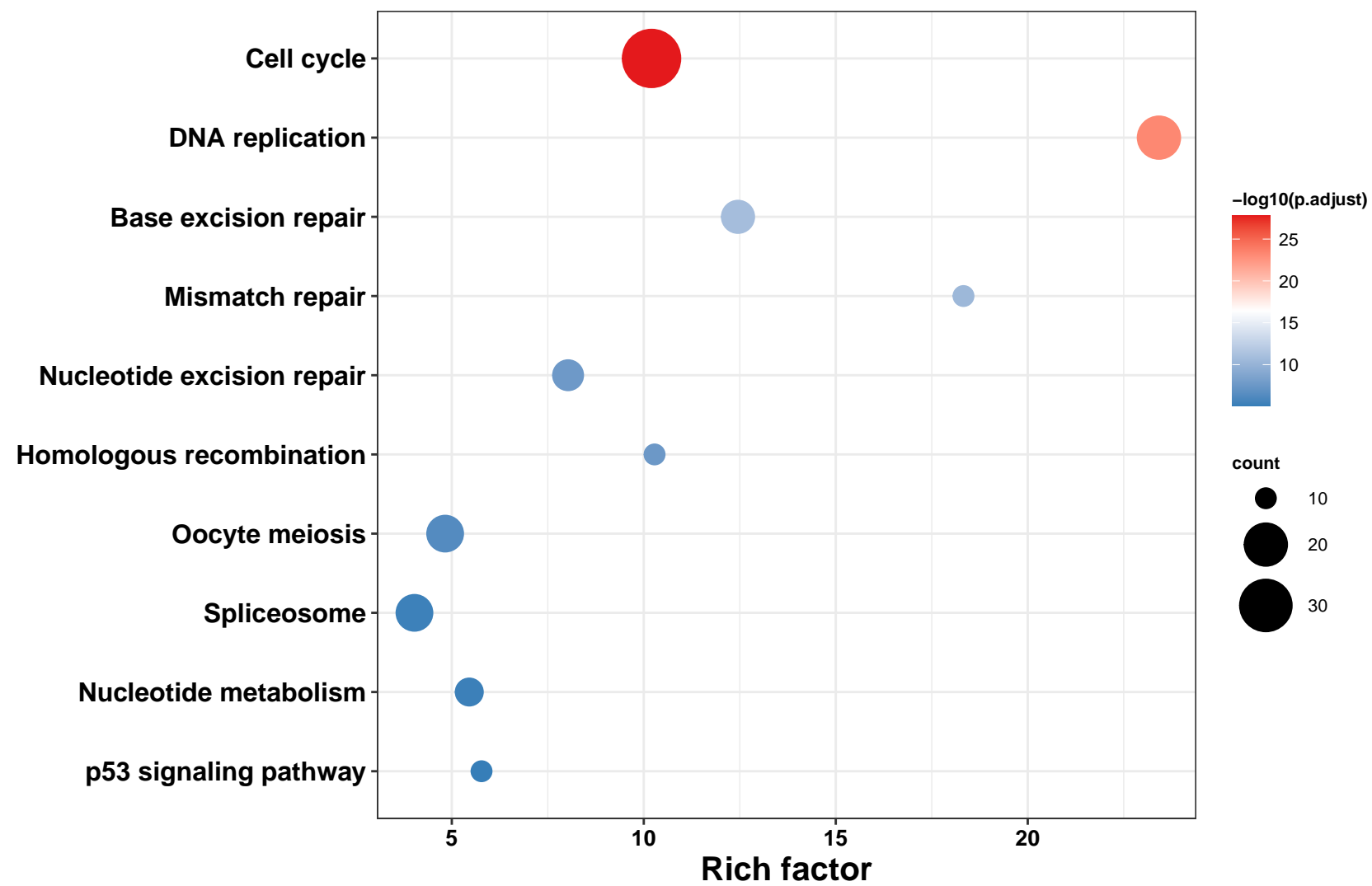

GO Biological Process

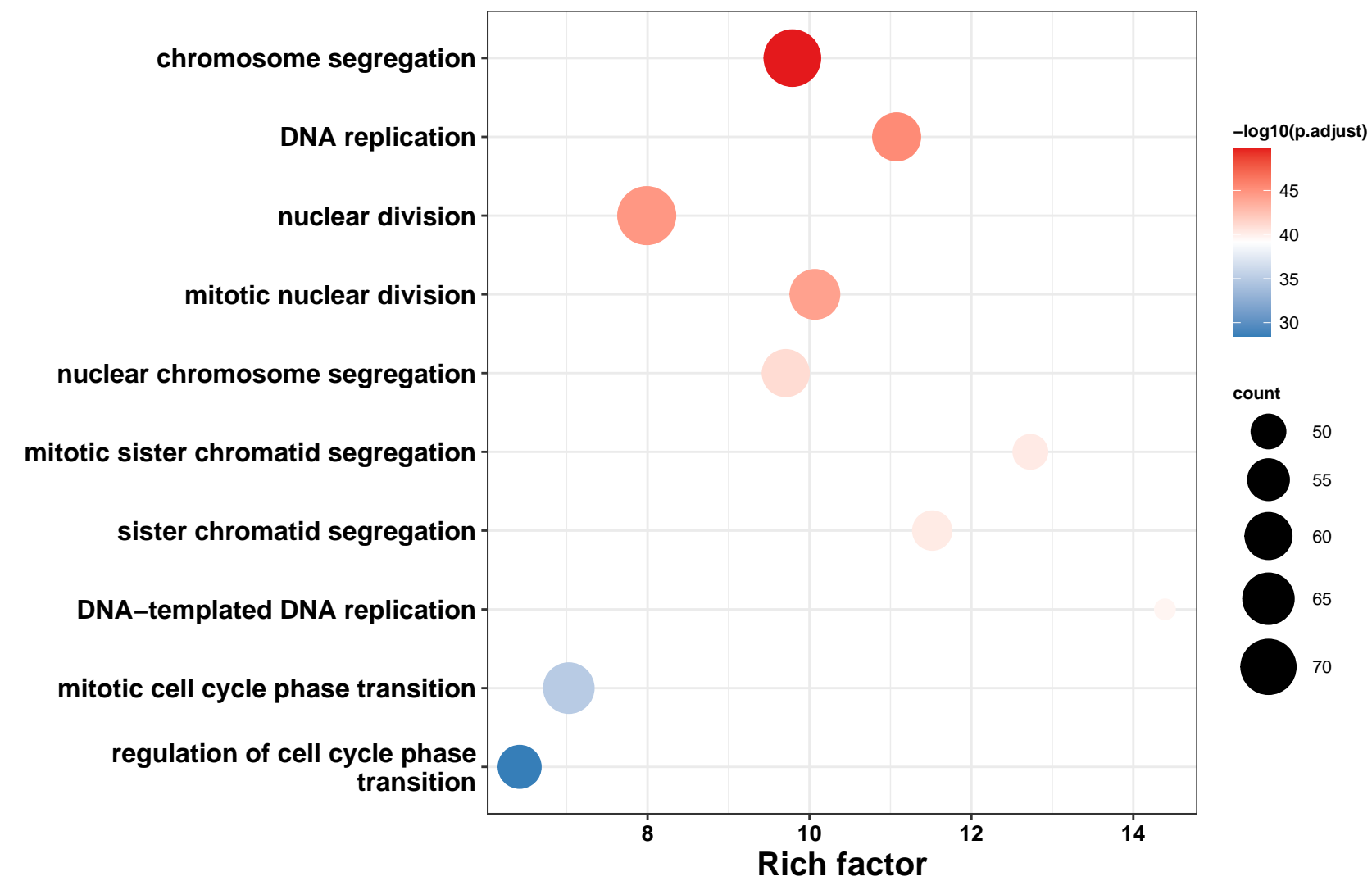

GO Molecular Function

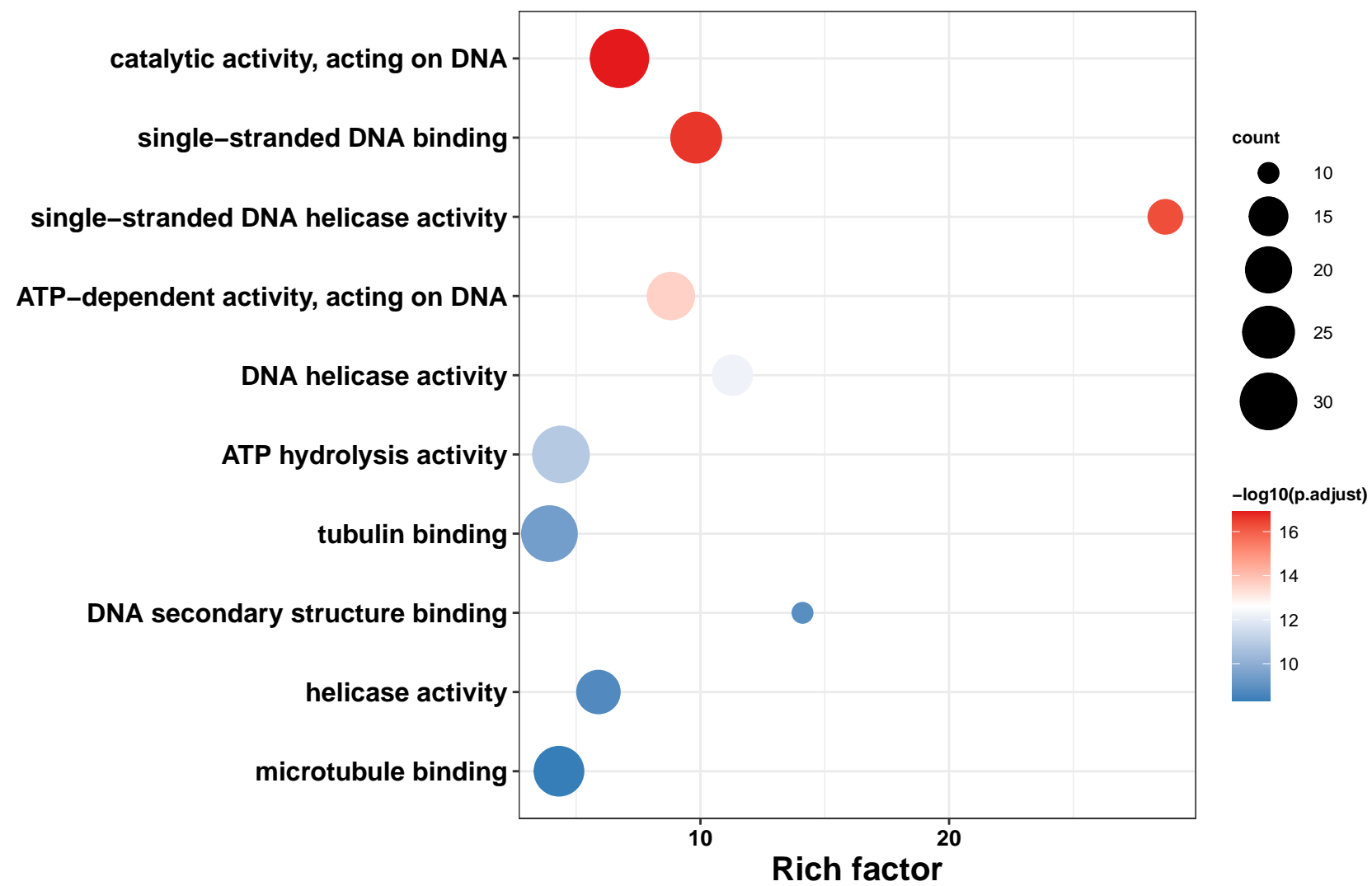

GO Cellular Component

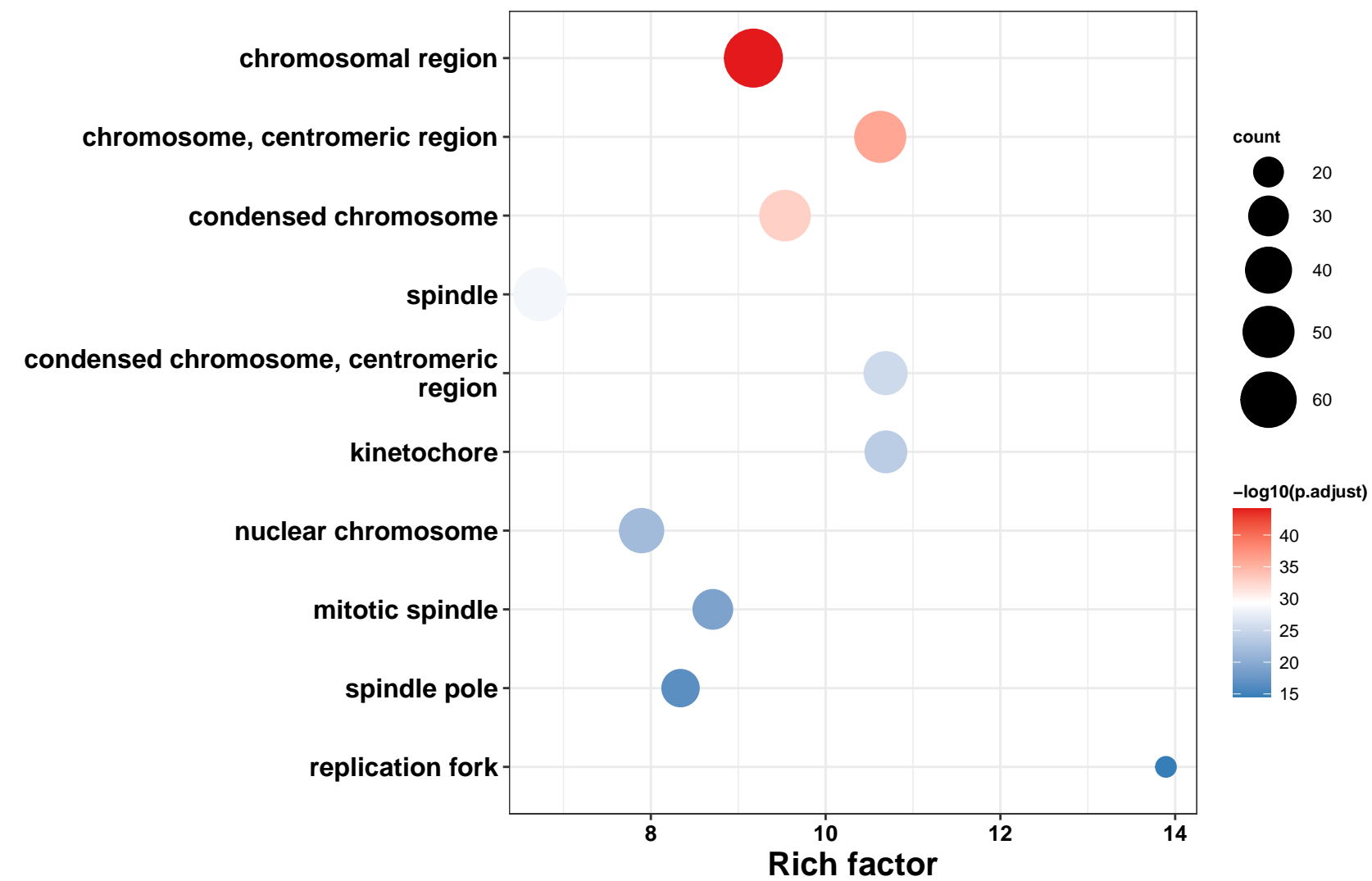

Supplement: Supplementary file 2 [file DataSheet4.pdf]

KEGG Pathway

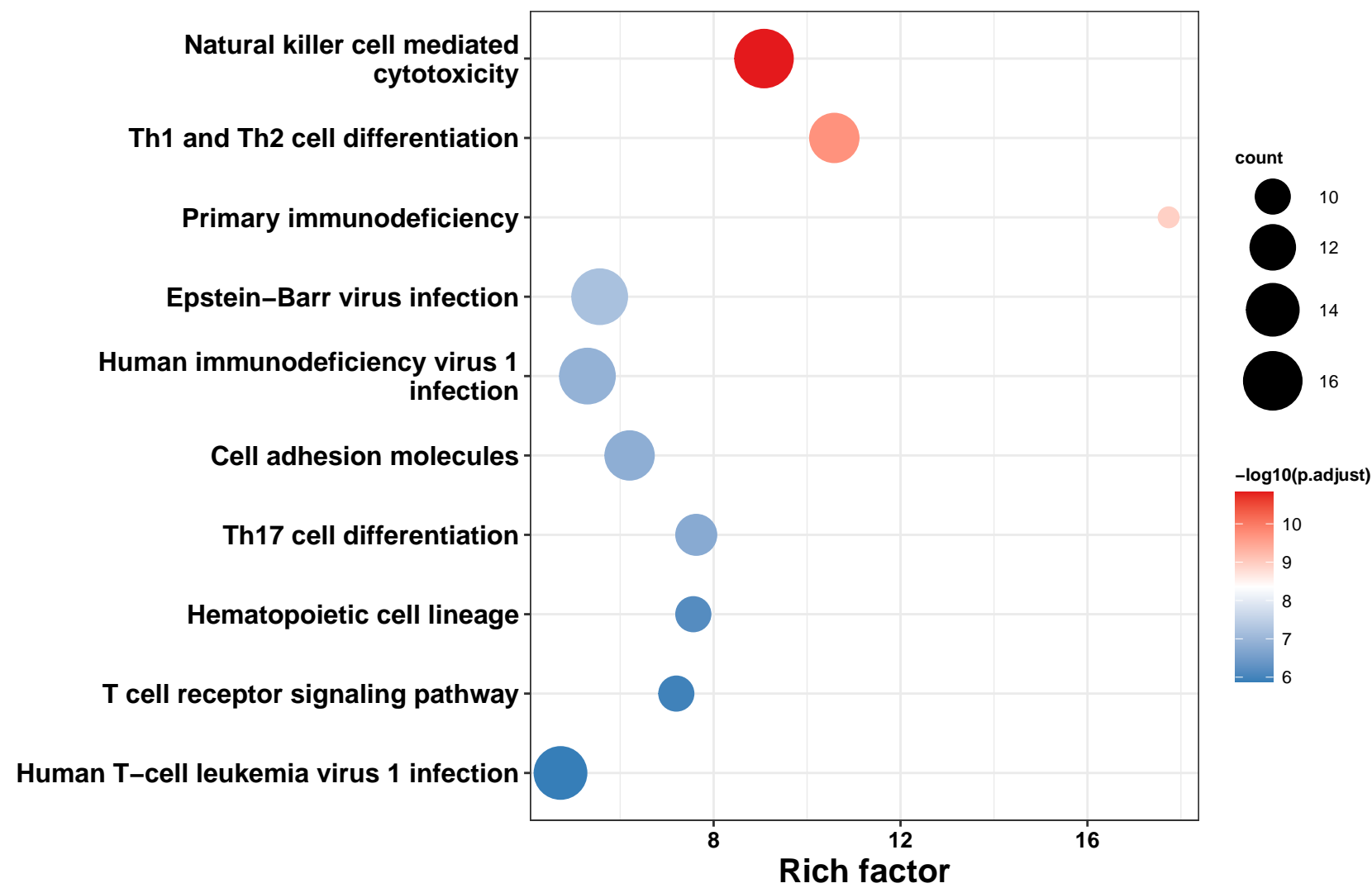

GO Biological Process

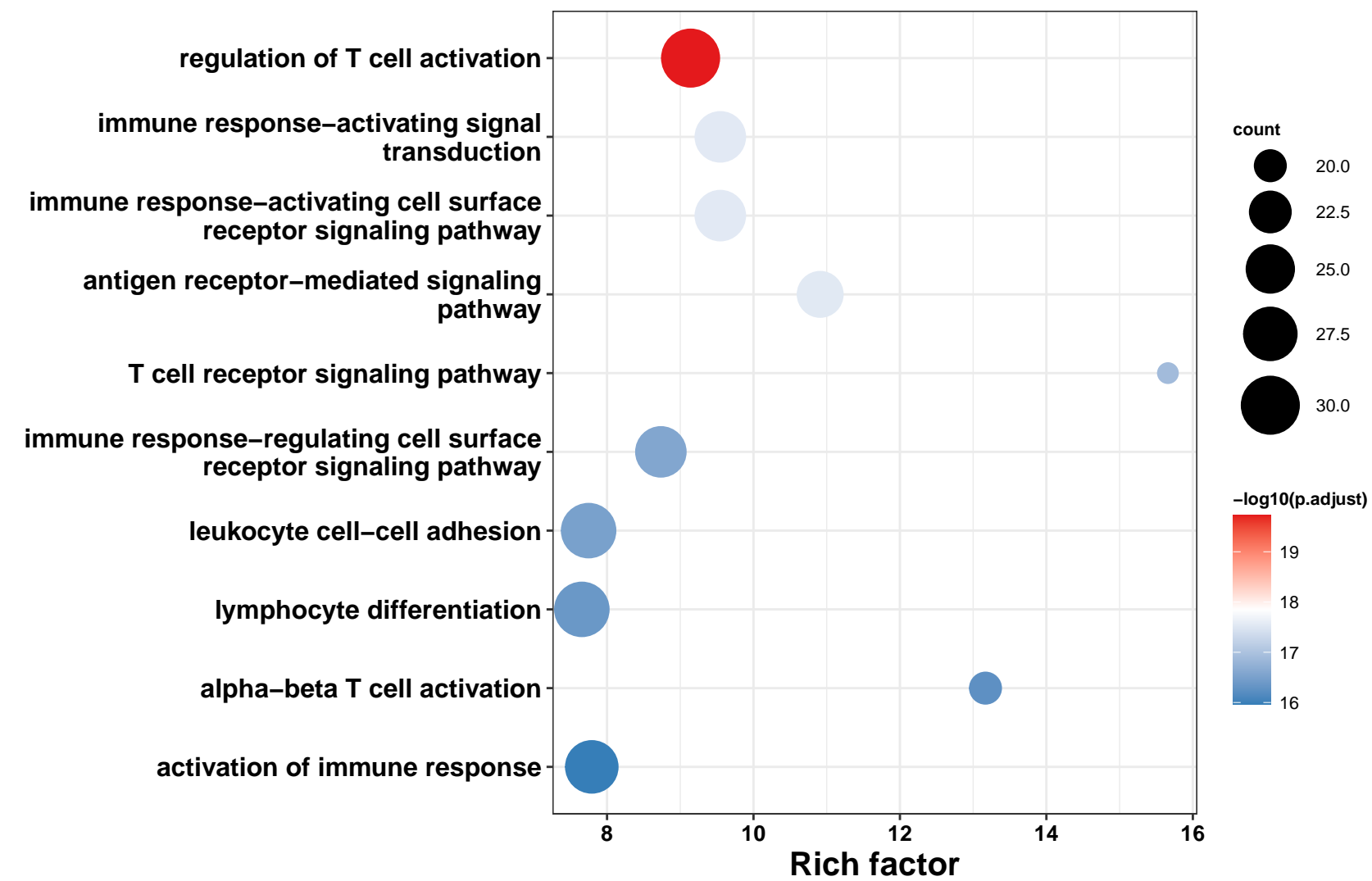

GO Molecular Function

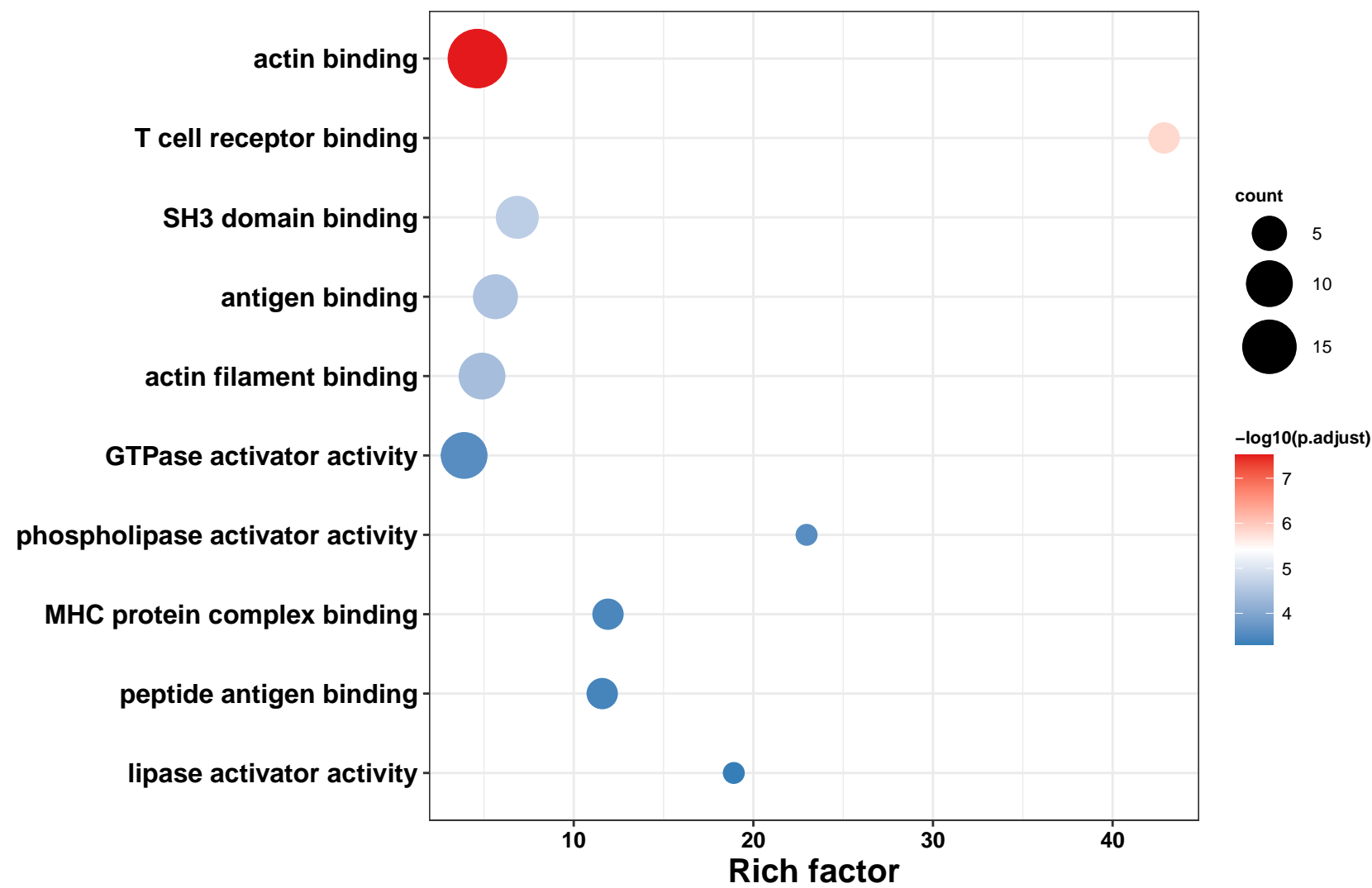

GO Cellular Component

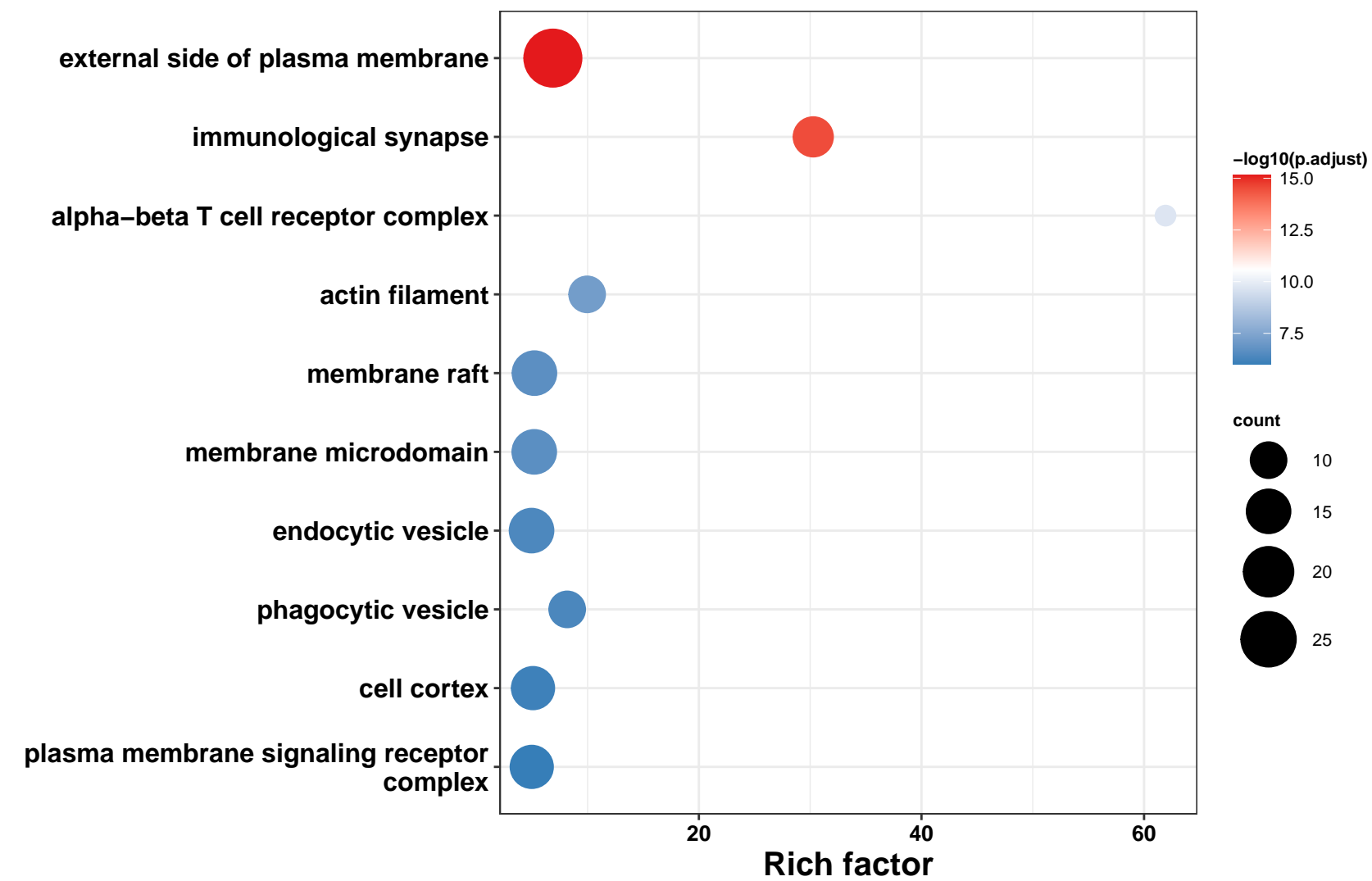

Supplement: Supplementary file 3 [file DataSheet3.pdf]

KEGG Pathway

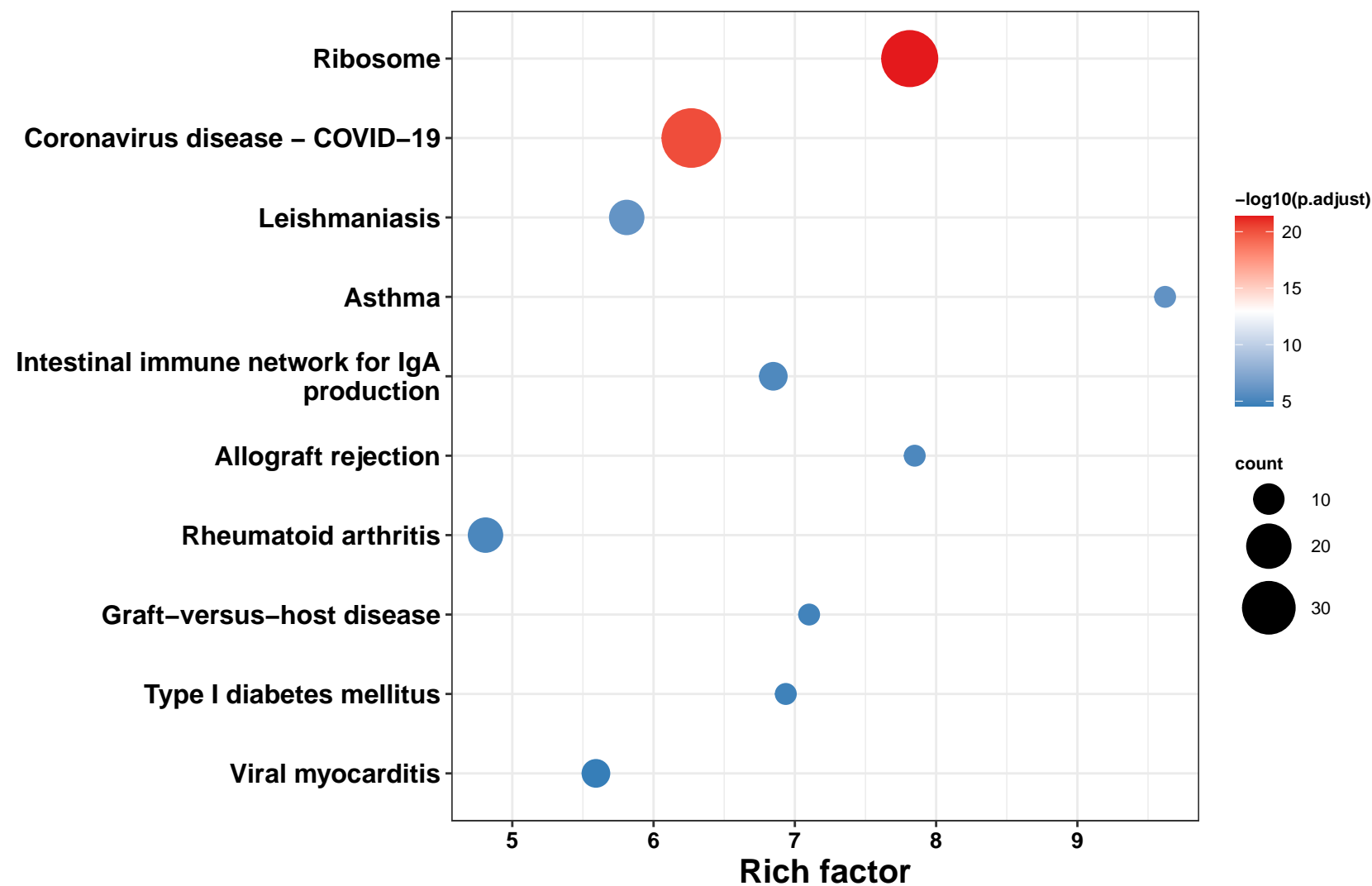

GO Biological Process

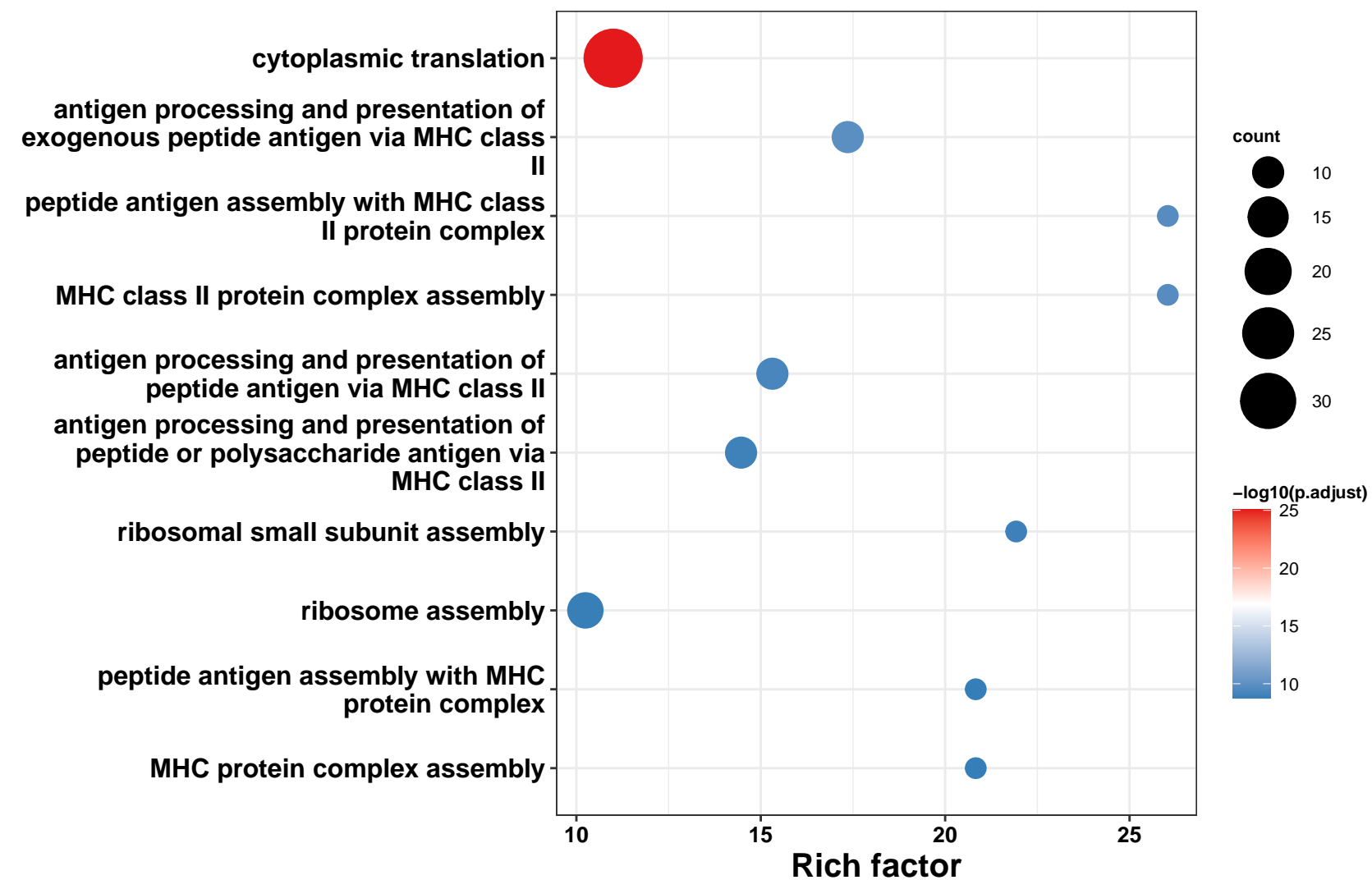

GO Molecular Function

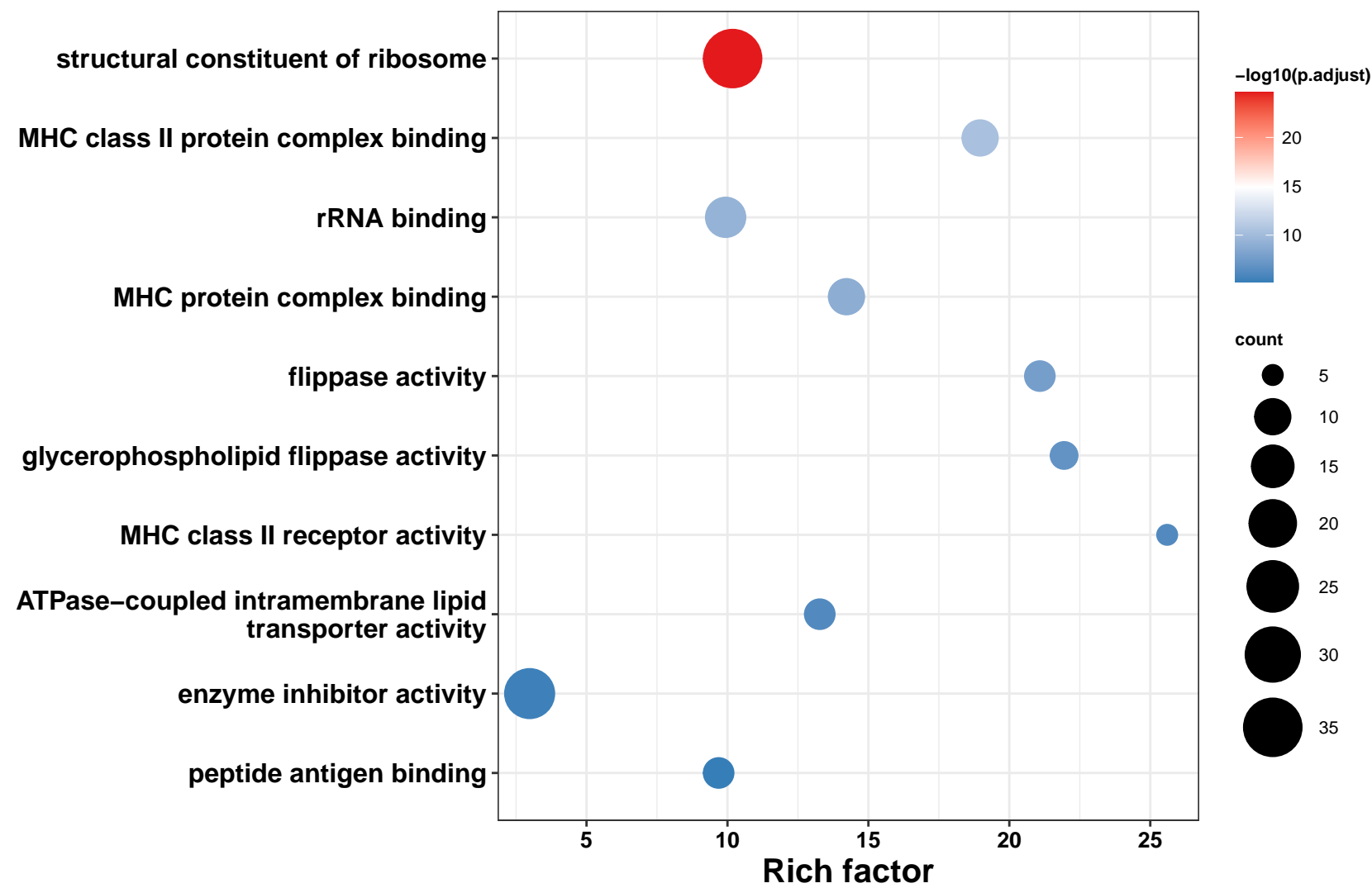

GO Cellular Component

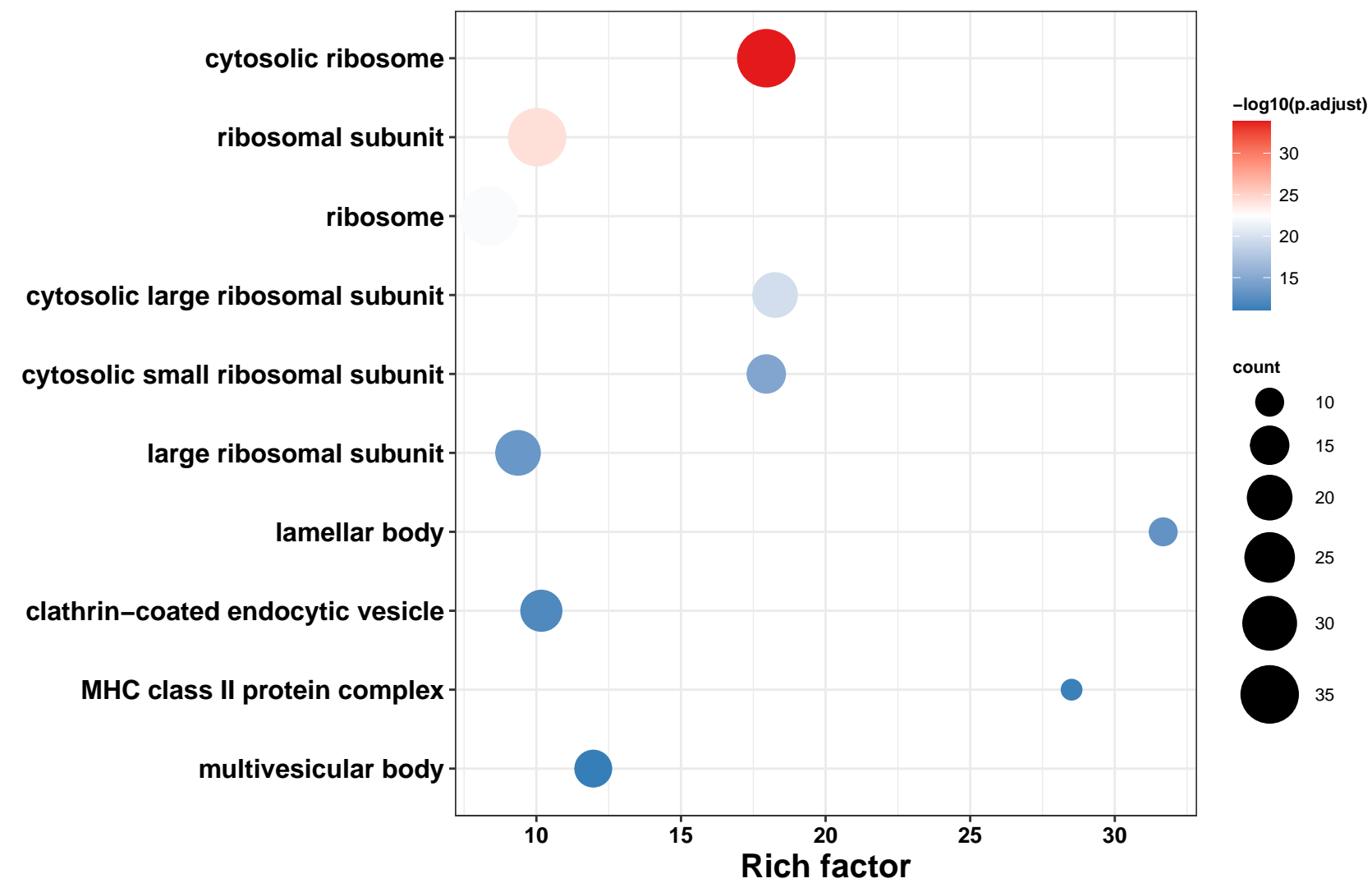

Supplement: Supplementary file 5 [file DataSheet1.pdf]

KEGG Pathway

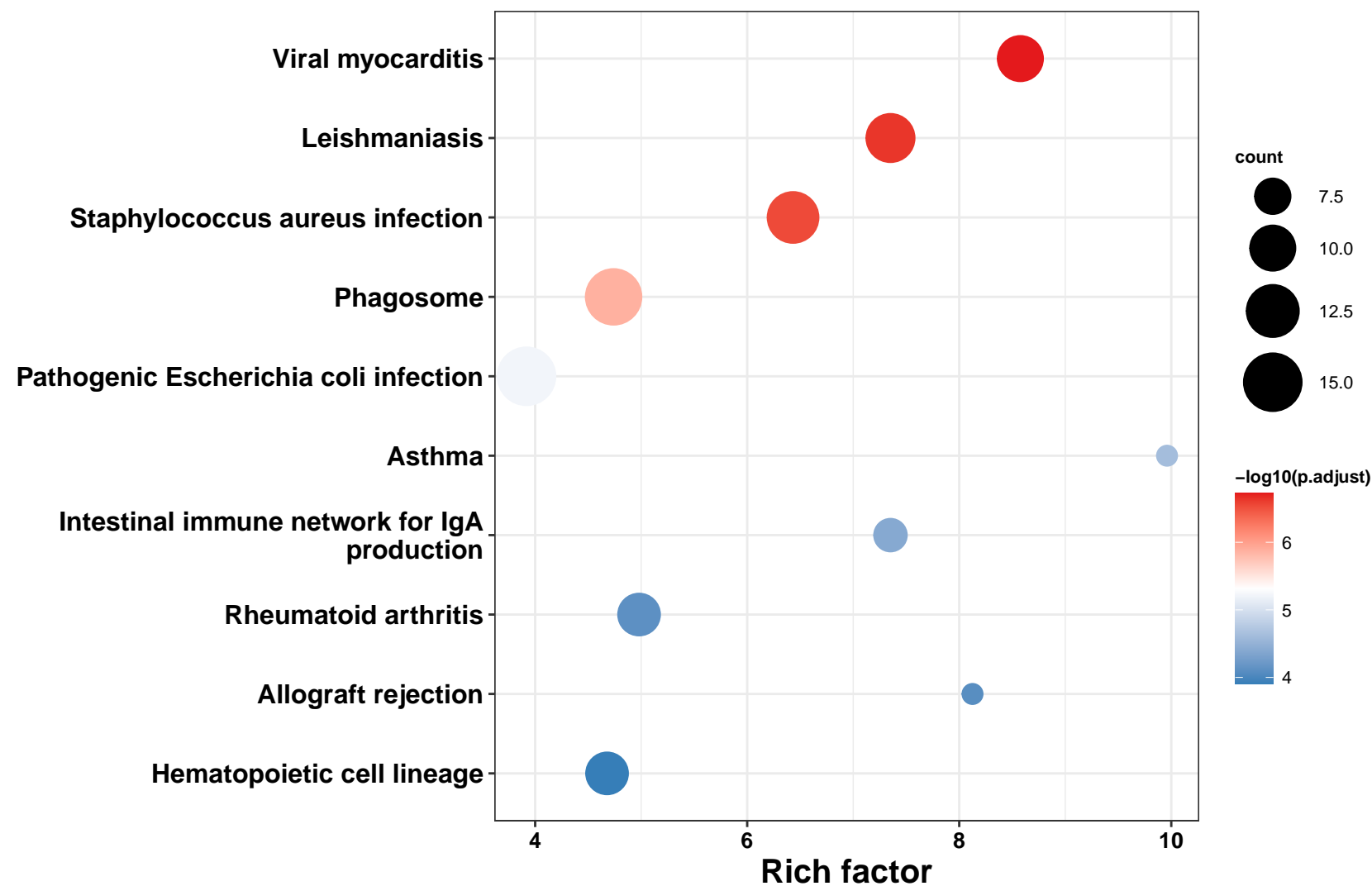

GO Biological Process

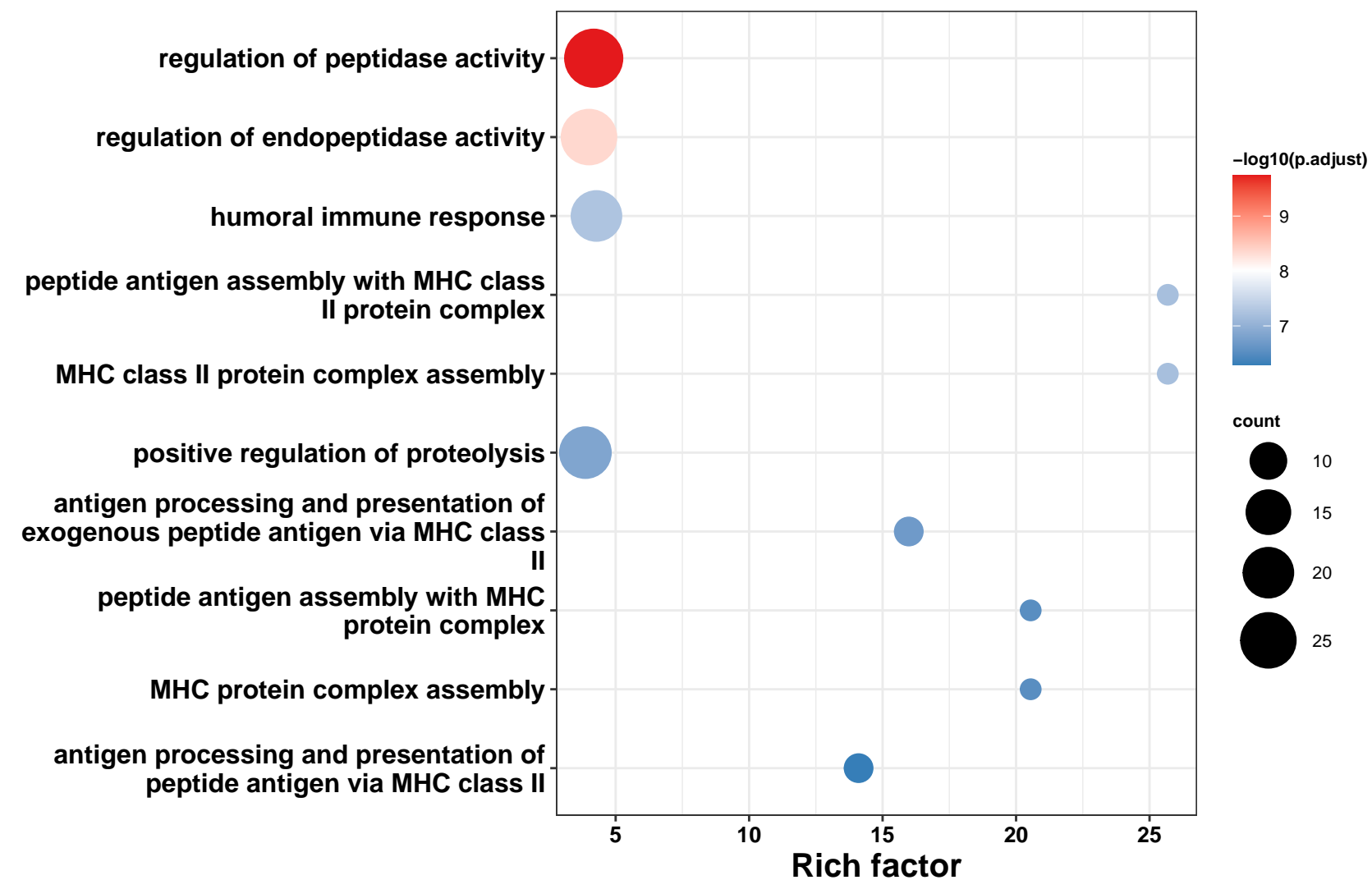

GO Molecular Function

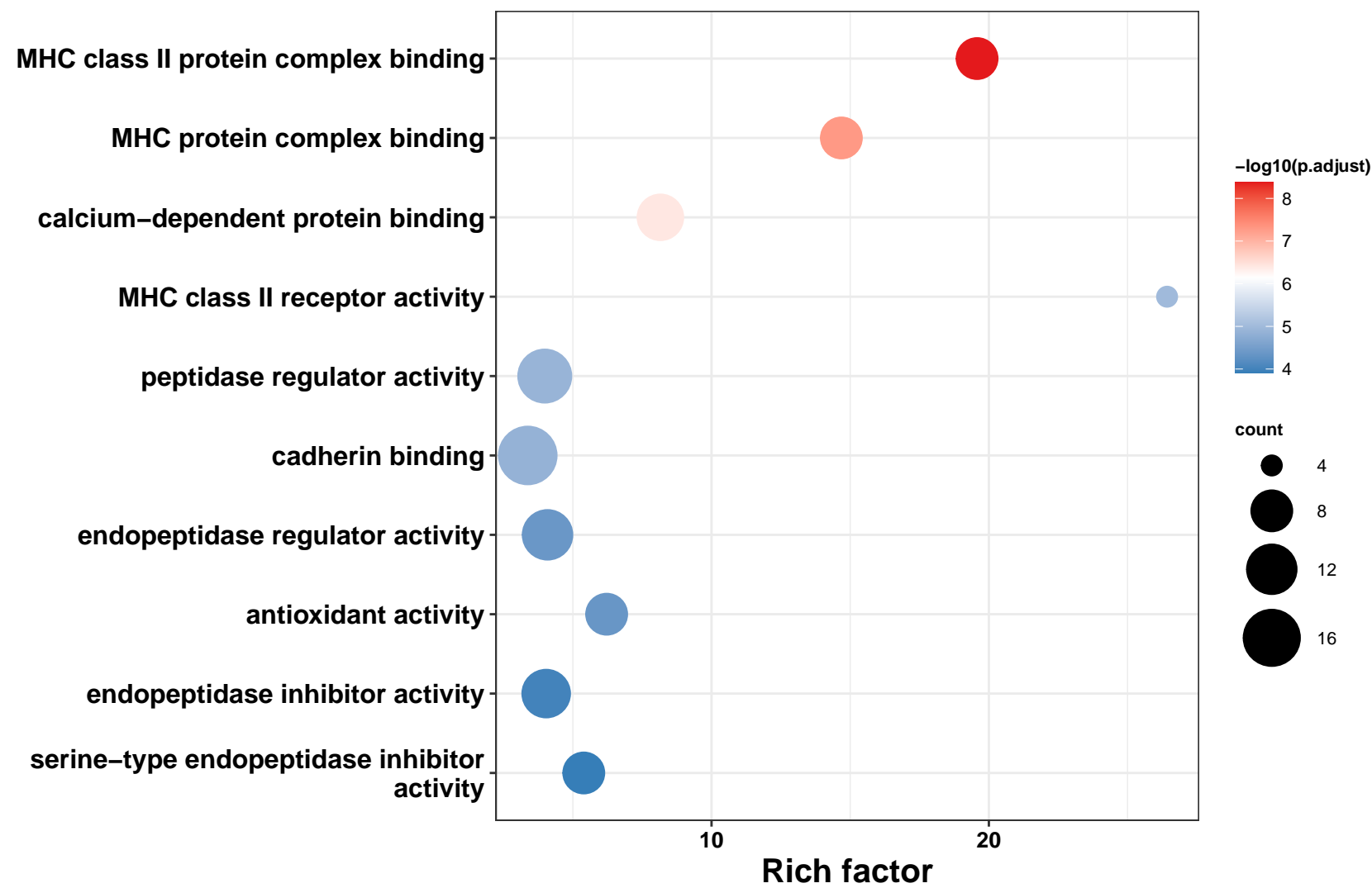

GO Cellular Component

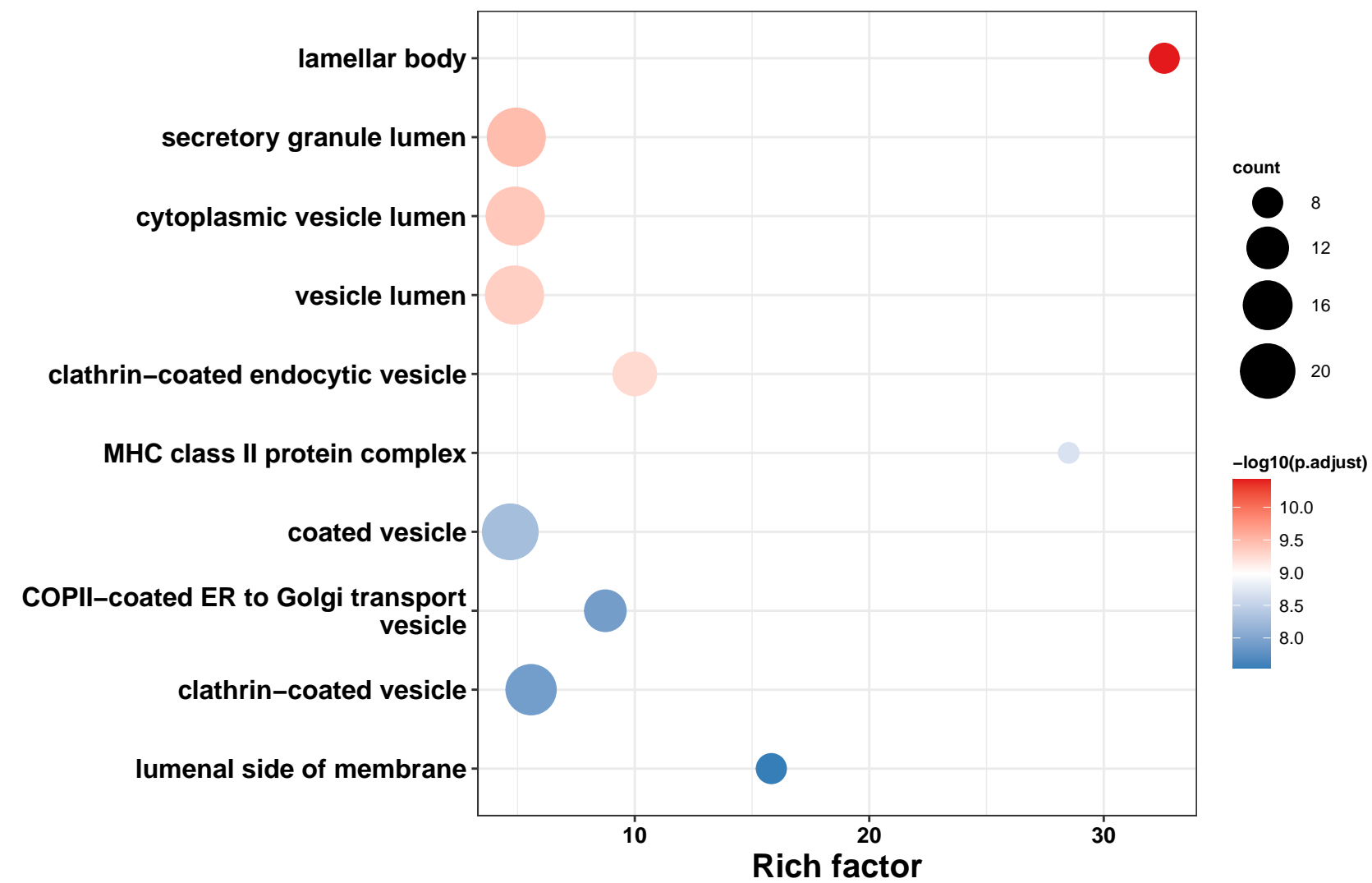

Supplement: Supplementary file 6 [file DataSheet5.pdf]
