# Supplementary material for: Integrated analysis of M2 macrophage-related gene prognostic model and single-cell sequence to predict immunotherapy response in lung adenocarcinoma
Source: Front Genet. 2025 Feb 3;16:1519677. doi: 10.3389/fgene.2025.1519677 (PMC11830816; doi:10.3389/fgene.2025.1519677)
Supplement: Supplementary file 4 [file Table2.docx]

Supplementary Information

Supplementary Tables

Supplementary Table 1. A total of 108 hub genes in the brown module in the database GSE26939.

Supplementary Figures

Fig. S1 Kyoto Encyclopedia of Genes and Genomes (KEGG) analysis and Gene Ontology (GO) analysis of over-expressed genes in the Sftpa1+mal subtype of lung cancer. Biological process (BP), molecular function (MF), and cellular component.

Fig. S2 Kyoto Encyclopedia of Genes and Genomes (KEGG) analysis and Gene Ontology (GO) analysis of over-expressed genes in the C15orf48+mal subtype of lung cancer. Biological process (BP), molecular function (MF), and cellular component.

Fig. S3 Kyoto Encyclopedia of Genes and Genomes (KEGG) analysis and Gene Ontology (GO) analysis of over-expressed genes in the Cxcr4+mal subtype of lung cancer. Biological process (BP), molecular function (MF), and cellular component.

Fig. S4 Kyoto Encyclopedia of Genes and Genomes (KEGG) analysis and Gene Ontology (GO) analysis of over-expressed genes in the Top2a+mal subtype of lung cancer. Biological process (BP), molecular function (MF), and cellular component.

Fig. S5 Kyoto Encyclopedia of Genes and Genomes (KEGG) analysis and Gene Ontology (GO) analysis of differentially expressed genes (DEG) between high- and low- cell groups. Biological process (BP), molecular function (MF), and cellular component.
